# CRISPR-Cas9 screen identifies oxidative phosphorylation as essential for cancer cell survival at low extracellular pH

**DOI:** 10.1016/j.celrep.2022.110493

**Published:** 2022-03-08

**Authors:** Johanna Michl, Yunyi Wang, Stefania Monterisi, Wiktoria Blaszczak, Ryan Beveridge, Esther M. Bridges, Jana Koth, Walter F. Bodmer, Pawel Swietach

**Affiliations:** 1 Department of Physiology, Anatomy and Genetics, Parks Road, Oxford OX1 3PT, UK; 2 Virus Screening Facility, MRC Weatherall Institute for Molecular Medicine, John Radcliffe Hospital, Oxford OX3 9DS, UK; 3 Department of NDM Experimental Medicine, MRC Human Immunology Unit, MRC Weatherall Institute of Molecular Medicine, JR Hospital, Headington, Oxford OX3 9DS, UK; 4 MRC Weatherall Institute for Molecular Medicine, John Radcliffe Hospital, Headington, Oxford OX3 9DS, UK

**Keywords:** tumor acidity, acidosis, CRISPR-Cas9 screen, oxidative phosphorylation

## Abstract

Unlike most cell types, many cancer cells survive at low extracellular pH (pHe), a chemical signature of tumors. Genes that facilitate survival under acid stress are therefore potential targets for cancer therapies. We performed a genome-wide CRISPR-Cas9 cell viability screen at physiological and acidic conditions to systematically identify gene knockouts associated with pH-related fitness defects in colorectal cancer cells. Knockouts of genes involved in oxidative phosphorylation (*NDUFS1*) and iron-sulfur cluster biogenesis (*IBA57*, *NFU1*) grew well at physiological pHe, but underwent profound cell death under acidic conditions. We identified several small-molecule inhibitors of mitochondrial metabolism that can kill cancer cells at low pHe only. Xenografts established from *NDUFS1*^−/−^ cells grew considerably slower than their wild-type controls, but growth could be stimulated with systemic bicarbonate therapy that lessens the tumoral acid stress. These findings raise the possibility of therapeutically targeting mitochondrial metabolism in combination with acid stress as a cancer treatment option.

## Highlights

• CRISPR screen identifies 51 genes required for survival at low versus physiological pH
• Cancer cell survival at low pH requires *NDUFS1* and other OXPHOS genes
• OXPHOS inhibitors selectively kill cancer cells at acidic pH, but allow survival at physiological pH
• *NDUFS1* knockout abrogates tumor xenograft growth in a pH-dependent manner

## Graphical abstract

**Figure:**
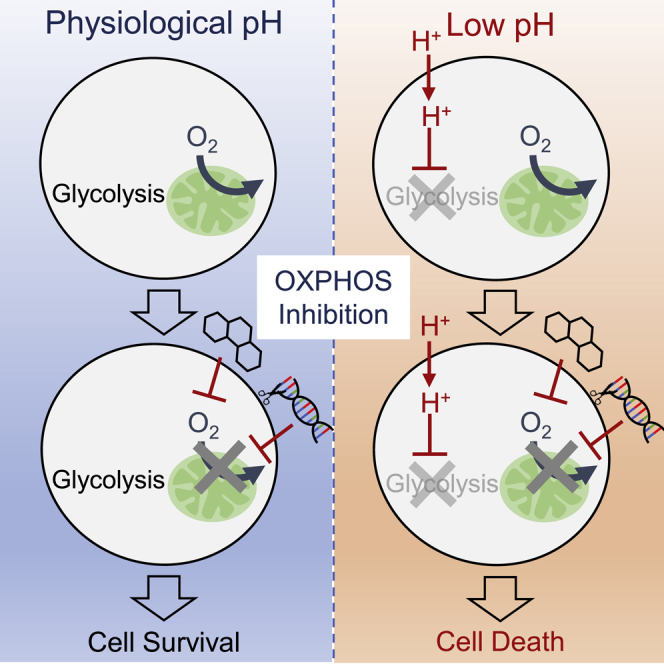

## Teaser

Alongside hypoxia, acidosis is a chemical signature of many solid tumors. By performing a genome-wide CRISPR-Cas9 screen, Michl et al. find that cancer cells require *NDUFS1* and other OXPHOS genes for survival in acidic environments. OXPHOS inhibitors selectively kill cancer cells at acidic pH, but permit survival at physiological pH, which defines a strategy for targeting acidic tumor regions.

## Introduction

Cancer cell proliferation, without its normal checks and controls, leads to a high metabolic rate. This is associated with intensified release of acidic end products, notably CO_2_ and lactic acid. As the tumor expands, capillary perfusion may become inadequate or unstable. This, in turn, leads to longer diffusion distances for solutes to travel. Upregulation of acid extrusion pathways further increase the extent of extracellular acidification (Boedtkjer and Pedersen, 2020). The juxtaposition of a high metabolic rate, increased acid extrusion, and diffusion-limited transport results in chemical gradients, most notably of H^+^ ions and of O_2_. The most recognizable feature of the tumor microenvironment is the emergence of acidotic and hypoxic regions. The pH/O_2_ landscape of tumors arises from the complex interplay between various metabolic pathways (Helmlinger et al., 1997; Vaupel et al., 1981; Rohani et al., 2019). Mitochondrial oxidative phosphorylation (OXPHOS) can occur even under restricted access to O_2_ in moderately hypoxic tissues (Fukuda et al., 2007); if, however, the energy harnessed by mitochondrial metabolism becomes inadequate, a fallback option is to enhance the glycolytic rate (Denko, 2008). Yet, this re-routing is self-limiting, as the accumulation of acidic products feeds back negatively on glycolysis (Corbet et al., 2014, Lamonte et al., 2013). As a result, the distribution of acidosis and hypoxia in a tumor is not necessarily overlapping. For instance, Rohani and colleagues (Rohani et al., 2019) have shown that acidosis can occur in normoxic regions, such as the tumor-stroma interface, whereas hypoxic conditions are more likely to be found at the tumor core. These observations suggest that tumor acidosis and hypoxia are maintained by distinct molecular pathways, and that cancer cells can be exposed to various combinations of acid and hypoxic stress.

The acidic tumor microenvironment (Gallagher et al., 2008) presents an unusual chemical milieu to cells otherwise adapted to survive at the physiological extracellular pH (pHe) of 7.4 (Webb et al., 2011). Unlike the case of hypoxia, for which sensors and targets are well-established (Denko, 2008; Jain et al., 2020), survival mechanisms under acidosis are less well characterized, not least because pH is more challenging to monitor and manipulate in a predictable manner (Michl et al., 2019). Although targeting tumor acidity is considered an excellent candidate for the therapeutic treatment of cancer (Neri and Supuran, 2011), so far none of the major approved tumor therapies are based explicitly on disrupting acid handling or signaling.

Extracellular acid stress may exert direct actions on proteins expressed at the cell surface or gain access to the intracellular compartment and therein influence a myriad of processes (Srivastava et al., 2007). This latter route arises from the coupling between pHe and intracellular pH (pHi). Glycolysis is an example of a pathway that is highly sensitive to pHi and, therefore, indirectly inhibited at low pHe. Although cancer cells commonly show the Warburg effect, the ensemble glycolytic rate will be reduced in acidic environments, potentially reaching critically low levels. Cancer cells must overcome these actions of acidity in order to survive. Once the necessary metabolic adaptation is implemented, cancer cells may exploit the benefits offered by low pHe, such as its pro-invasive properties (Peppicelli et al., 2014; Kato et al., 1992). Previous studies have described that cancer cells adapt to acidity through an increase in glutamine metabolism, OXPHOS, and fatty acid metabolism (Corbet and Feron, 2017). Acidity can also cross over to hypoxic signaling by inhibiting HIF-1α and activating HIF-2α, driving glutamine uptake and metabolism (Corbet et al., 2014). Low pHe also promotes fatty acid synthesis and oxidation through the downregulation of acetyl-CoA carboxylase 2 (Corbet et al., 2016). However, these observations do not necessarily identify the genes that confer a survival advantage under acidosis; instead, they may be epiphenomena or consequences in a hierarchy of secondary responses. A more definitive test would require a comprehensive and unbiased gene knockout (KO) screen. This approach would identify specific KOs that change survival under acid stress. Once validated, such genes would offer a targeted means of exploiting acid adaptation as a therapeutic approach to treating cancer.

Here, we performed a systematic screen by introducing Toronto KO (TKO) v.3 library containing guide RNAs (sgRNAs) targeting >18,000 protein-coding genes into the SW480 colorectal cancer cell line. Mutant cells were treated with media at physiological, mildly acidic, or highly acidic pHe (7.4, 6.9, and 6.6, respectively). We then compared the frequency of sgRNAs in cells surviving under these acid-base conditions to identify genes associated with survival at low or high pHe. Using this unbiased approach, we identified metabolic processes that are required for cell proliferation under acid stress. In particular, components of the OXPHOS pathway and iron-sulfur cluster biogenesis were found to be essential for survival in an acidic environment. These genes represent suitable therapeutic targets for cancer therapies. Based on our findings, we reasoned that their inhibition would selectively reduce the survival prospects of cancer cells in acidic regions, without affecting well-perfused, normal tissues in an interstitium of physiological pHe.

## Results

### Genome-wide CRISPR-Cas9 screen identifies genes essential for survival under low and physiological pHe

**Figure 1:**
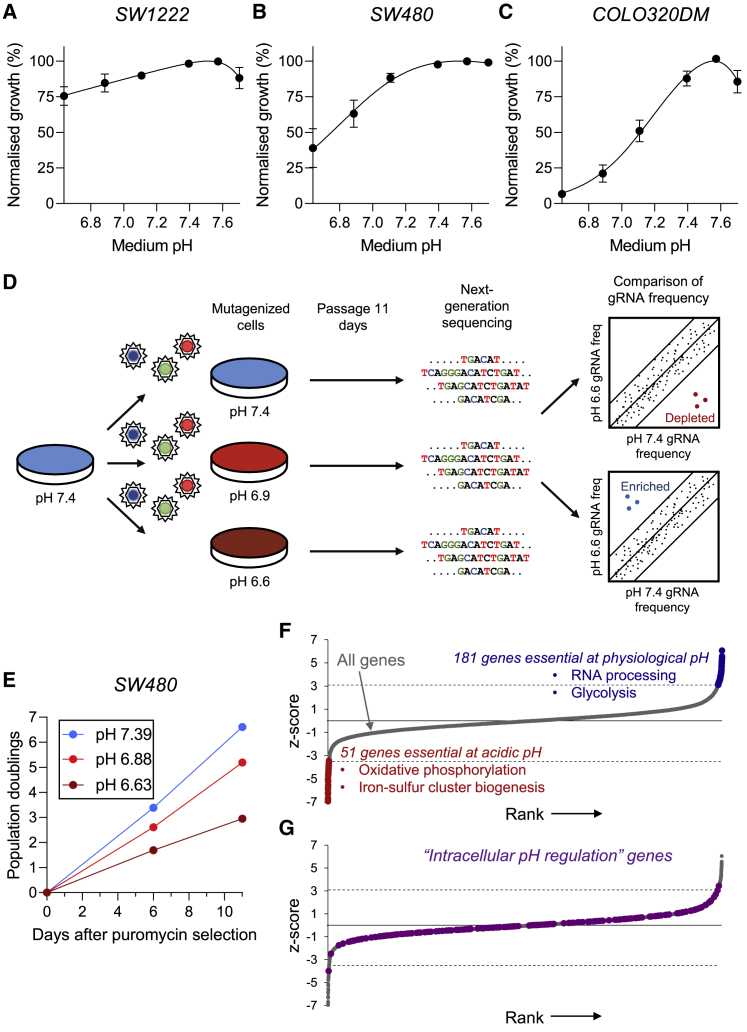
Whole-genome CRISPR-Cas9 cell viability screen identifies genes underpinning acid sensitivity and acid resistance (A–C) Cell growth reported over a range extracellular pH (pHe) in SW1222, SW480, and COLO320DM cells, measured using SRB absorbance (mean ± SEM of five independent repeats, with three technical replicates each). (D) Scheme of experimental design for genome-wide screen. Three medium pHe conditions were tested at day 11 of treatment (two independent repeats). (E) Cumulative proliferation of cells at pHe 6.6, 6.9, 7.4 (mean of n = 2). (F) All 18,049 genes are ranked by their selective essentiality at pHe 6.6 versus pHe 7.4. Red and blue color indicates indicate significantly depleted and enriched genes at 10% false discovery rate (FDR). Dashed line represents threshold level of 10% FDR. (G) Genes of the Gene Ontology “intracellular pH regulation” pathway (extended with additional pHi-regulating genes) highlighted as purple circles along the overall ranking of all genes, as in (F). Dashed line represents threshold level of 10% FDR.

To identify specific genes that are essential for survival under acidic conditions, we performed a genome-wide CRISPR-Cas9 screen and then validated the highest-ranking hits on a case-by-case basis. This experimental workflow involved three colorectal cancer (CRC) cell lines (SW1222, SW480, and COLO320DM), selected on the basis of their pHe sensitivity of growth. To define the pHe sensitivity, cells were cultured at low density for 6 days in media of predefined pHe, set by adjusting [HCO_3_^–^] to a concentration between 0 and 44 mM at 5% CO_2_. Growth at the endpoint was determined from biomass by sulforhodamine B (SRB) assay (Figures 1A–1C). The emergent survival curves determined the level of pH at which growth is halved relative to growth at the optimum pHe (pH_50_). SW1222 cells were found to be relatively pH insensitive, while SW480 had attenuated growth at modestly acidic conditions, and COLO320DM manifested a steeper pH sensitivity. Extracellular acidification is recognized to have a knock-on effect on intracellular pH (pHi), and a difference in this trans-membrane coupling may contribute toward the contrasting responses of CRC cells to low pHe. To test this, the pHe-pHi relationship was determined in cells loaded with the pH reporter cSNARF1 and equilibrated over the pHe range studied (Figure S1). SW1222, SW480, and COLO320DM cells manifested a very similar pHi-pHe relationship. The positive slope of this relationship indicates that pHi falls under acidic conditions and can then evoke powerful actions on intracellular processes. However, the pHe-pHi relationships were similar in all three CRC lines, which indicates that additional mechanisms are responsible for enabling survival at low pHe in specific lines, such as SW1222.

Being the intermediate phenotype, SW480 cells were selected for the CRISPR-Cas9 screen. Subsequent validation experiments were performed on SW480 cells and either SW1222 (for genes conferring acid resistance) or COLO320DM (for genes conferring acid sensitivity).

We generated a genome-wide pool of SW480 knockout cells using the TKO v.3 library, targeting 18,049 protein-coding genes using 70,948 sgRNAs (using four sgRNAs per gene). Following puromycin selection, we divided the cells into three populations for culture in media at physiological pHe (7.4), moderately acidic pHe (6.9), or highly acidic pHe (6.6) (Figure 1D), prepared by adjusting [HCO_3_^–^] (22 mM–2.75 mM) at constant (5%) CO_2_. Surviving cell populations were collected after 11 days of culture. To ensure near constancy of pHe throughout the experiment, cells were sub-cultured to avoid over-confluence and the media were replaced every 3 days. Cell growth was monitored throughout the screening process. As expected, cells cultured under acidic conditions grew considerably slower than those cultured at physiological pHe (Figure 1E).

At the end of the pHe adaptation process, cells were collected for next-generation sequencing of integrated sgRNA sequences. Quality control, read counts, and normalization of sequencing data were performed using the MAGeCK Flute pipeline. Subsequent analysis using the DrugZ pipeline assigned *Z* scores to genes that are enriched under one of the conditions studied. This approach identified gene KOs that were differentially abundant in cells cultured at low versus physiological pHe. We identified 51 gene KOs that were significantly depleted (*Z* < −3.51) at pHe 6.6 relative to pHe 7.4, and 181 gene KOs that are significantly enriched (*Z* > 3.09) in pHe 6.6 relative to pHe 7.4, taking a false discovery rate (FDR) of 0.1 (Figure 1F and Table S1 and S2). Gene enrichment analysis identified the electron transport chain and OXPHOS as essential at a highly acidic pHe of 6.6 (Table S3). Related to these mitochondrial processes, genes involved in iron-sulfur cluster biogenesis were also found to be essential for survival under low pHe. In contrast, genes involved in RNA processing, telomere maintenance, and canonical glycolysis were determined to be selectively essential at physiological pHe (Table S3). Of note, gene enrichment scores were generally higher for genes deemed essential at acidic pHe, which indicates a good degree of confidence in those hits.

### Individual genes of the “intracellular pH regulation pathway” are not essential for survival at low pHe

Under the premise that most pH-sensitive proteins are located intracellularly, we reasoned that pHi regulators are plausible candidates for essential genes under acidic conditions. This pathway includes genes coding for membrane transporters of H^+^ and HCO_3_^–^ ions, inferred to give a growth benefit to cancer cells on the basis of the actions of various small-molecule drugs and inhibitory antibodies (Corbet and Feron, 2017). Surprisingly, most pHi-regulating genes were not among the highest-ranking hits identified in our screen (Figure 1G). The only exception was *SLC9A1*, which encodes for the sodium-hydrogen exchanger 1 (NHE1) but it ranked only 33^rd^ among genes found to be essential under acidic conditions. Notable pHi-regulating genes, including *SLC16A1*, coding for the H^+^-monocarboxylate transporter 1, and *SLC4A7* encoding the electroneutral Na^+^/HCO_3_^–^ co-transporter were not significantly enriched in either physiological nor acidic conditions. In interpreting these findings, it is important to consider the redundancy among pHi regulators, such that the knockout of one gene may be compensated by the activity of other genes coding for proteins with a similar transport function.

### Genes involved in mitochondrial metabolism are essential at low pHe

**Figure 2:**
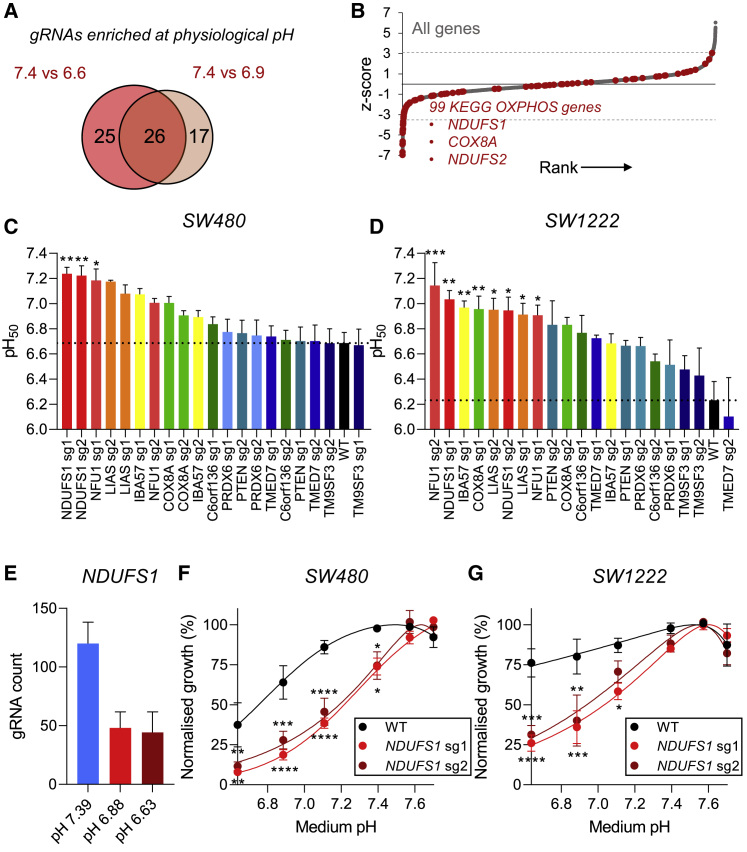
Genes involved in mitochondrial metabolism are essential for survival under acidic conditions (A) Venn diagram showing the number of genes that are essential under mildly acidic (pHe 6.9) and highly acidic (pHe 6.6) conditions, indicating the degree of overlap. (B) KEGG OXPHOS genes highlighted in red along the overall ranking of genes, as in Figure 1F. Dashed line represents threshold level of 10% FDR. (C and D) Experimental validation of screen hits in SW480 and SW1222 KO cell lines. Growth was assayed at day 6 as a function of pHe (mean ± SEM of three to four independent repeats, with three technical replicates each). pH_50_ value represents the pHe at which growth is halved relative to that at the optimum pHe. pH_50_ values for wild-type cells represent average values obtained from different batches of experiments. Dotted line indicates pH_50_ value of non-transduced wild-type cells. (E) sgRNA abundance at different pHe levels of the screen for the *NDUFS1* gene coding for a complex I subunit. Mean relative abundance (± SEM) shown across four guides per gene across two screen replicates. (F and G) Normalized growth rates (measured by SRB absorbance) at 6 days as a function of pHe of wild-type, *NDUFS1* sg1-infected, and *NDUFS1* sg2-infected SW480 and SW1222 cell pools. Data are plotted as relative cell growth normalized to optimum pHe (mean ± SEM of three to four independent repeats, with three technical replicates each). Significance determined with two-way ANOVA using Šídák's multiple comparisons test (^∗^p < 0.05, ^∗∗^p < 0.01, ^∗∗∗^p < 0.001; ns, non-significant, p > 0.05).

We next compared genes identified as essential for survival under conditions that are mildly acidic (pHe 6.9; 43 genes; Table S4) or highly acidic (pHe 6.6; 51 genes; Table S1). Although the analysis of these two datasets was undertaken independently, a high degree of overlap became apparent among the hits. Twenty-six genes were significantly depleted in both mildly and highly acidic culture conditions (Figure 2A). The majority of the highest-ranking genes deemed essential for survival under highly acidic conditions related to mitochondrial metabolism (Figure 2B). These included genes coding for subunits of complex I (*NDUFS1*, *NDUFS2*, *NDUFA11*) and complex VI (*COX8A*) of the electron transport chain. Other significantly enriched KOs related to iron-sulfur cluster biogenesis (*NFU1* and *IBA57*), which is required for complex I function. Next, we sought to validate these findings on SW480 cells and the more acid-resistant SW1222 line. Lentiviral infected cell pools with individual sgRNAs (Table S6) were generated by puromycin selection. The surviving pools were tested for the pHe sensitivity of growth measured by SRB assay on day 6. We tested 10 individual gene KOs, each using two different sgRNA sequences, and determined the pH_50_ values (pH_50_ values lower than the most acidic pHe tested [pHe 6.63] were extrapolated by curve fitting). For wild-type cells, pH_50_ values were averaged from the different experimental batches. Three of the tested sgRNAs produced a significant increase in pH_50_ (i.e., becoming more acid sensitive) compared with non-transduced wild-type cells of the acid-sensitive SW480 line (Figure 2C). In contrast, eight of the tested sgRNAs led to a significant increase in pH_50_ in the more acid-resistant SW1222 line (Figure 2D). Overall, *NDUFS1* and *NFU1* gRNAs caused SW1222 and SW480 cells to become more pHe sensitive, and these genes were selected for further studies.

sgRNA counts for *NDUFS1* were highly enriched under alkaline screening conditions, and significantly depleted at both mildly and highly acidic pHe (Figure 2E). Consistent with this observation, infection with individual *NDUFS1* sgRNAs led to a significant increase in pH_50_ values in both SW480 and SW1222 cells (Figures 2F and 2G). Similar effects were observed with KOs for lipoic acid synthetase (*LIAS*), iron-sulfur cluster assembly factor IBA57 (*IBA57*), and NFU1 iron-sulfur cluster scaffold (*NFU1*). It is noteworthy that several tested sgRNAs also reduced growth at physiological pHe, but the effect was more pronounced under acidic conditions (Figures S2 and S3).

Taken together, these findings suggest that mitochondrial metabolism pathways are essential for cell growth under acidic conditions. Mechanistically, this can be explained in terms of the profound inhibition of glycolysis at low pHe, leaving cells more reliant on mitochondrial procurement of energy. Targeting genes such as *NDUFS1*, *NFU1*, or *IBA57* is therefore a potential route for selectively killing cancer cells in acidic microenvironments.

### Glycolysis is selectively essential at physiological pH but dispensable at acidic pHe

**Figure 3:**
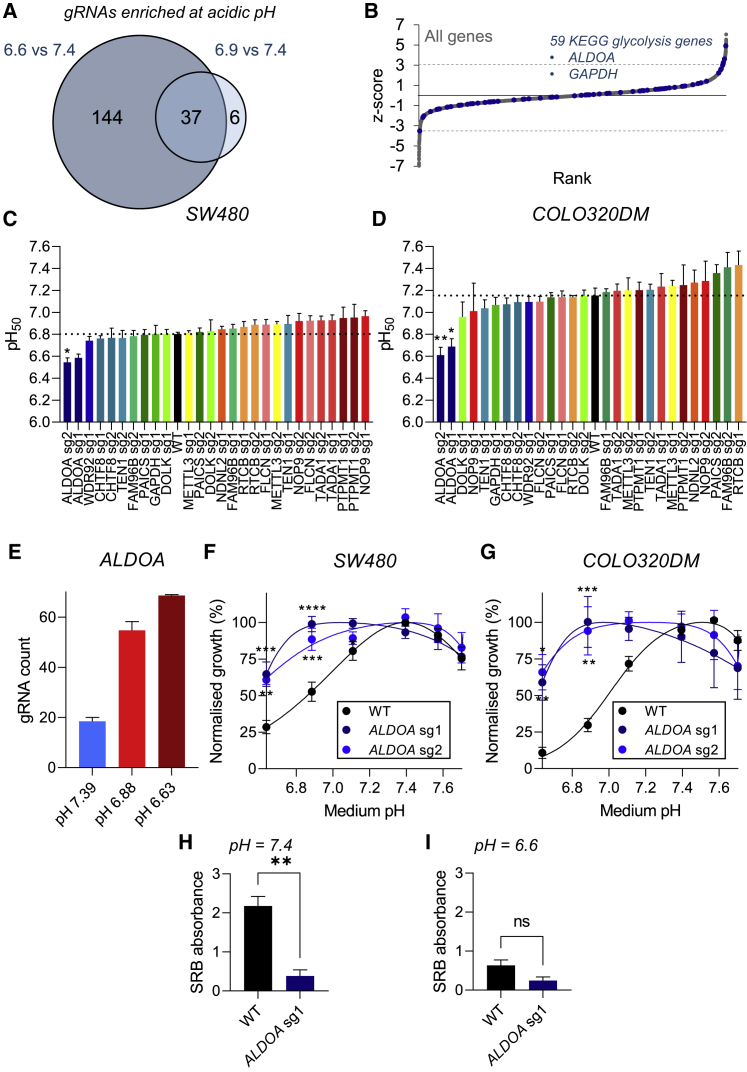
Glycolysis is essential at physiological pHe but dispensable at low pHe (A) Venn diagram showing number of gene knockouts enriched in mildly acidic (pHe 6.9) and highly acidic (pHe 6.6) conditions, indicating the degree of overlap. (B) KEGG glycolysis genes highlighted in red along the overall ranking of genes, as in Figure 1F. Dashed line represents threshold level of 10% FDR. (C and D) Experimental validation in SW480 and COLO320DM KO cell lines. Growth was assayed at day 6 as a function of pHe (mean ± SEM of three independent repeats, with three technical replicates each). pH_50_ value represents the pHe at which growth is halved relative to that at the optimum pHe. pH_50_ values for wild-type cells represent average values obtained from different batches of experiments. Dotted line indicates pH_50_ value of non-transduced wild-type cells. gRNAs for the same gene are shown with a unique color. (E) sgRNA abundance at different pHe levels of the screen for *ALDOA*. Mean relative abundance (± SEM) shown across four guides per gene across two screen replicates. (F and G) Normalized growth rates at 6 days as a function of pHe (measured by SRB absorbance) of wild-type, *ALDOA* sg1-infected, and *ALDOA* sg2-infected SW480 or COLO320DM cell pools. Data are plotted as relative cell growth normalized to optimum pHe (mean ± SEM of three independent repeats, with three technical replicates each). Significance determined with two-way ANOVA using Šídák's multiple comparisons test. (H and I) Absolute cell growth (measured by SRB absorbance) in wild-type and *ALDOA* sg1-infected SW480 cells cultured for 6 days at pHe = 7.4 or pHe = 6.6 (mean ± SEM of three independent repeats, with three technical replicates each). Significance determined with two-tailed unpaired t test (^∗^p < 0.05, ^∗∗^p < 0.01, ^∗∗∗^p < 0.001; ns, non-significant, p > 0.05).

A number of genes involved in RNA splicing, telomere maintenance, and glycolysis were among highly ranked KOs enriched under acidic conditions. A central theme connecting these pathways to survival at physiological pHe is less apparent. Moreover, there was also less overlap (only 37 genes) between KOs enriched at mildly acidic (43 genes; Table S5) and highly acidic conditions (181 genes; Figure 3A; Table S2). Notably, however, genes involved in canonical glycolysis were enriched within these gene sets (Figure 3B). Validation experiments were performed on SW480 cells and the more acid-sensitive cell line COLO320DM. Polyclonal cell populations were prepared by infection with lentiviral vectors containing individual sgRNAs, followed by puromycin selection. In most cases, the transduction of either cell line with sgRNAs did not lead to a significant change in pHe sensitivity of growth (Figures 3C and 3D, S4, and S5). Only the inactivation of fructose-bisphosphate aldolase A (*ALDOA*) resulted in a significant decrease in pH_50_ in both SW1222 and COLO320DM cells, consistent with the notion that glycolysis is inhibited under acidic conditions (Chen et al., 2008; Corbet et al., 2014, Lamonte et al., 2013). In terms of sgRNA count, *ALDOA* showed a dose-dependent enrichment at low pHe screening conditions in SW480 cells (Figure 3E). This finding indicates that *ALDOA* is not needed for optimal growth under acidic conditions. Strikingly, infection with individual *ALDOA* sgRNAs led to a more acid-resistant phenotype in both SW480 and COLO320DM cells (Figures 3F and 3G). It is noteworthy that *ALDOA* inactivation in SW480 cells caused a profound reduction in absolute cell numbers at physiological pHe (Figure 3H). However, the effect was not statistically significant at low pHe (Figure 3I).

### Acidosis leads to reduced glycolysis and increased production of reactive oxygen species

**Figure 4:**
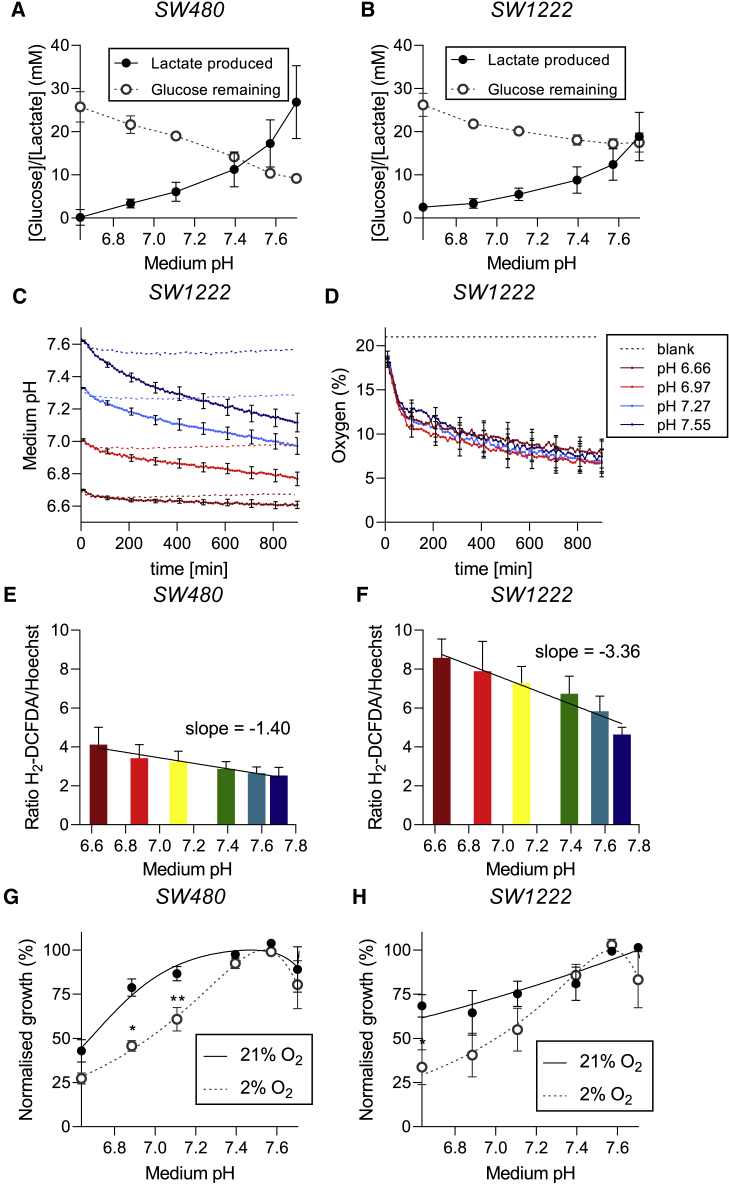
Low pHe suppresses glycolysis and stimulates the production of reactive oxygen species (A and B) Relationship between lactate production and glucose consumption (measured by biochemical assay) as a function of pHe in SW480 and SW1222 cells (mean n = 3 independent repeats ± SEM). (C and D) Time courses of medium pH and O_2_ as a function of pHe for SW1222 wild-type cells. pHe and O_2_ were measured using HPTS and RuBP fluorescence, respectively, in media buffered with 10 mM HEPES and 10 mM MES. (mean n = 4 independent repeats, carried out in technical triplicate). (E and F) Reactive oxygen species (ROS) levels in SW480 and SW1222 cells cultured for 6 days at varying pHe. ROS levels expressed as H_2_DCFDA fluorescence normalized to Hoechst 33342 fluorescence (mean ± SEM of five independent repeats, with three technical replicates each). (G and H) Normalized growth (measured by SRB absorbance) of SW480 and SW1222 cells cultured for 6 days at 21% O_2_ versus 2% O_2_. Data are plotted as relative cell growth normalized to optimum pHe (mean ± SEM of three to four independent repeats, with three technical replicates each). Significance determined with two-way ANOVA using Šídák's multiple comparisons test (^∗^p < 0.05, ^∗∗^p < 0.01, ^∗∗∗^p < 0.001; ns, non-significant, p > 0.05).

Since several genes related to mitochondrial pathways were among the highest-ranking hits in our screen, we tested whether cells cultured in acidic conditions become more dependent on these pathways. Based on biochemical measurements performed on media after 6 days of culture at different pHe, we found that SW1222 and SW480 cells produced less lactate at low pHe, compared with cells grown in alkaline medium (Figures 4A and 4B). Consistent with this, glucose consumption increased with rising pHe. Using a real-time fluorimetric assay to measure changes in pHe and pO_2_ in media at constant buffering capacity, we found that SW1222 cells acidified their media only from a high starting pHe (Figure 4C). This confirms that glycolysis is strongly inhibited under acidic conditions, and therefore the genes belonging to this pathway should not be essential for survival at low pHe. In contrast, O_2_ consumption persisted even at very low pHe (Figure 4D). Since previous studies linked elevated OXPHOS with higher levels of reactive oxygen species (ROS), we tested ROS levels using H_2-_DCFDA fluorescence after exposure of cells to an acidic medium. A negative correlation between ROS (normalized to cell density using Hoechst-33342) and pHe was observed in both SW480 and SW1222 cells (Figures 4E and 4F). Interestingly, ROS levels were, overall, higher in SW1222 compared with SW480 cells, suggesting an inherent preference for OXPHOS-based metabolism in SW1222 cells, which may relate to its acid-resistant phenotype. Since OXPHOS function requires O_2_, we tested whether culturing under hypoxia would influence the cell's pHe sensitivity. Cells cultured at 2% O_2_ for 6 days had decreased growth under alkaline and acidic conditions, compared with cells in 21% O_2_ (Figures 4G and 4H). The growth defect was, however, more pronounced over the acidic range of pHe. This is consistent with the notion that fully operational mitochondrial activity is necessary for cell growth under acidic conditions.

### *NDUFS1* knockout cells have impaired survival under acidic conditions

**Figure 5:**
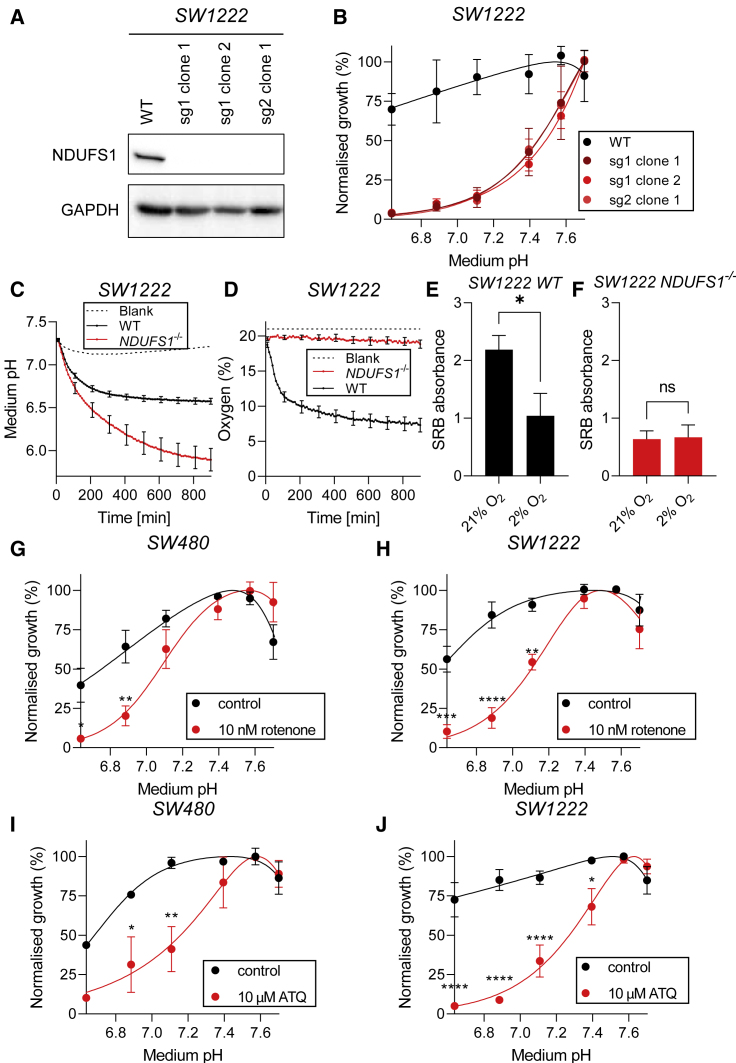
Inhibition of OXPHOS selectively kills cancer cells cultured under acidic conditions (A) Western blot of lysates from SW1222 wild-type and *NDUFS1*^−/−^ clones. (B) Normalized growth rates (measured by SRB absorbance) of SW1222 wild-type and *NDUFS1*^−/−^ clonal cell lines cultured for 6 days. Data are plotted as relative cell growth normalized to optimum pHe (mean ± SEM of three independent repeats, with three technical replicates each). Significance determined with two-way ANOVA using Šídák's multiple comparisons test. (C and D) Time courses of medium pH and O_2_ as a function of pHe for SW1222 wild-type and *NDUFS1*^−/−^ cells. pHe and O_2_ were measured using HPTS and RuBP fluorescence, respectively, in media buffered with 2 mM HEPES and 2 mM MES. cells (mean ± SEM of eight independent repeats, with six technical replicates each). (E and F) Absolute cell growth (measured by SRB absorbance) in WT and *NDUFS1*^−/−^ cells cultured for 6 days at 21% O_2_ versus 2% O_2_ at pHe 7.7 (mean ± SEM of six independent repeats, with three technical replicates each). Significance determined with two-tailed unpaired t test. (G and J) Normalized growth rates (measured by SRB absorbance) of SW480 and SW1222 and cells cultured for 6 days with 10 nM rotenone, 10 μM atovaquone (ATQ), or vehicle. Data are plotted as relative cell growth normalized to optimum pHe (mean n = 3–4 independent repeats ± SEM; carried out in technical triplicates). Significance determined with two-way ANOVA using Šídák's multiple comparisons test (^∗^p < 0.05, ^∗∗^p < 0.01, ^∗∗∗^p < 0.001; ns, non-significant, p > 0.05).

To further test whether inhibition of complex I function could be a viable strategy for selectively killing cancer cells under acidic conditions, we established a clonal SW1222 *NDUFS1*^−/−^ cell line (Figure 5A). Three clones were obtained, all of which showed a growth defect under acidic conditions (Figure 5B). Growth of *NDUFS1*^−/−^ cells decreased as pHe was reduced, until proliferation became completely inhibited at pHe 6.6. The absence of this complex I subunit could lead to increased production of ROS due to incomplete electron transfer to molecular oxygen. We found increased ROS levels in *NDUFS1*^−/−^ cells at acidic pHe, which may be responsible for the cell death (Figure S6). We also observed that *NDUFS1*^−/−^ cells generated considerably greater quantities of lactic acid than wild-type cells (Figure 5C), which indicates an exacerbated reliance on glycolysis. In contrast, oxygen consumption in *NDUFS1*^−/−^ cells was blocked completely, whereas wild-type cells depleted medium of O_2_ over the same time-frame (Figure 5D). Further evidence for metabolic differences between *NDUFS1*^−/−^ and wild-type cells was sought from their responses to hypoxia. At pHe 7.7, absolute cell growth of wild-type cells was reduced after 6-day culture at 2% O_2_, compared with 21% O_2_ (Figure 5E). In contrast, growth of *NDUFS1*^−/−^ cells was largely insensitive to ambient O_2_ levels (Figure 5F).

### Pharmacological inhibition of OXPHOS selectively kills cells under acidic conditions

In light of the striking consequences of genetically ablating OXPHOS genes on the cell's pHe sensitivity of growth, we tested whether pharmacological inhibition has comparable efficacy to KO. We treated SW480 and SW1222 cells with low concentrations of rotenone, a lipophilic selective inhibitor of complex I (Heinz et al., 2017). At 10 nM, rotenone did not result in significant cytotoxicity when probed at pHe 7.7, but it became a potent inhibitor of proliferation at pHe 6.6 (Figures 5G and 5H). This drug is not, however, suitable for therapeutic applications owing to its toxicity (Sherer et al., 2003) and Parkinsonian-like side effects in humans (Patel, 2011). We therefore tested other small-molecule inhibitors of the mitochondrial electron transport chain with fewer documented toxicological concerns. Atovaquone (ATQ) is an antimalarial drug that inhibits mitochondrial complex III and increases tumor oxygenation in a range of cell lines (Ashton et al., 2016) as well as non-small cell lung cancer patients (Skwarski et al., 2021). In SW480 and SW1222 cells, ATQ led to a striking reduction in growth under acidic conditions (Figures 5I and 5J). Other inhibitors of the electron transport chain, including piericidin A, deguelin, and the type II diabetes mellitus drug metformin, had similar actions to ATQ (Figure S7). The efficacy of these drugs at blocking OXPHOS was confirmed by performing measurements of O_2_ consumption (Figure S7). Previous studies have shown that adaptation of cancer cells to chronic acidosis can affect gene expression (Yao et al., 2020), and this may, in turn, influence responses to OXPHOS inhibitors. To test this, the effects of rotenone or ATQ were measured in SW480 and SW1222 cells that had been adapted for 1 week at pH 6.3 (titrated by reducing [HCO3^–^] and supplemented with 10 mM MES to provide additional buffering). The growth response to these OXPHOS inhibitors was not changed in acid-adapted cells, relative to time-matched cells kept at physiological pH (Figure S8).

To test whether OXPHOS is important for non-cancer cell survival at low pHe, experiments were performed on intestinal fibroblasts and CCD18 colonic fibroblasts (Figure S8). Compared with most cancer cell lines, these cells are more reliant on mitochondrial metabolism and therefore have a shallow pHe dependence. However, growth of myofibroblasts became inhibited at low pHe in the presence of ATQ or rotenone, and a similar observation was noted for CCD18 cells treated with ATQ. Thus, the role of OXPHOS activity in enabling growth in acidic conditions also applies to non-cancer cells.

Taken together, we demonstrate that inhibiting OXPHOS by appropriately titrated pharmacological drugs is a strategy for selectively killing cancer cells in acidic microenvironments.

### Iron-sulfur cluster biogenesis is essential for survival under acidic conditions

**Figure 6:**
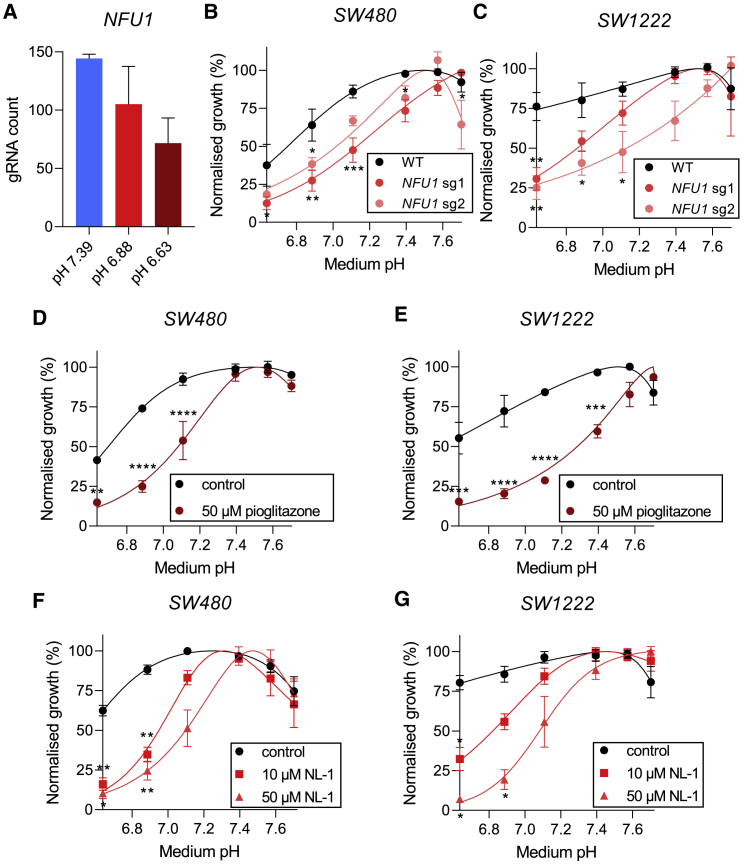
Iron-sulfur cluster biogenesis is essential for survival under acidic conditions (A) sgRNA abundance at different time pHe levels of the screen for *NFU1*. Mean relative abundance (± SEM) shown across four guides per gene across all screen replicates. (B and C) Normalized growth rates (measured by SRB absorbance) of wild-type, *NFU1* sg1-infected, and *NFU1* sg2-infected cell pools. Data are plotted as relative cell growth normalized to optimum pHe (mean ± SEM of three to four independent repeats, with three technical replicates each). (D and E) Normalized growth rates (measured by SRB absorbance) of SW480 and SW1222 and cells cultured for 6 days with 50 μM pioglitazone or vehicle. Data are plotted as relative cell growth normalized to optimum pHe (mean ± SEM of three independent repeats, with three technical replicates each). Significance determined with two-way ANOVA using Šídák's multiple comparisons test (^∗^p < 0.05, ^∗∗^p < 0.01, ^∗∗∗^p < 0.001; ns, non-significant, p > 0.05).

In addition to the molecular components of the electron transport chain, we found that genes involved in iron-sulfur cluster biogenesis (*NFU1*, *IBA57*) were among the highest ranked genes determined to be essential for survival under acidic conditions. From the results of the screen on SW480 cells, sgRNA counts for *NFU1* were reduced at both mildly and highly acidic pHe, compared with alkaline conditions (Figure 6A). Validation of these findings using two individual sgRNAs confirmed that *NFU1* was required by SW480 and SW1222 cells for survival under acidic conditions (Figures 6B and 6C). We then tested whether pharmacological inhibition of iron-sulfur cluster biogenesis would result in comparable effects on cell growth under acidic conditions. The mitoNEET inhibitor pioglitazone, used in the treatment of type 2 diabetes mellitus, reduced growth selectively at low pHe in both SW480 and SW1222 cells (Figures 6D and 6E). This may be due to pioglitazone's effect on stabilizing the 2Fe-2S cluster release from the outer mitochondrial membrane (Patel, 2011). This finding was confirmed using another mitoNEET blocker, NL-1, which dose dependently reduced growth of SW480 and SW1222 cells selectively at low pHe (Figures 6E and 6F). We next tested whether depleting iron as a substrate for iron-sulfur cluster biogenesis would have to similar effects on the cell's pHe sensitivity. Surprisingly, chelating iron with deferiprone or deferoxamine (Figure S9) did not result in a steeper pHe sensitivity, but this may relate to the plethora of other drug actions unrelated to iron-sulfur clusters.

Given that iron-sulfur cluster biogenesis is essential for cell survival under acidic conditions, we tested whether the growth defect at low pHe could be rescued by supplementing media with iron in the form of iron(II) sulfate. Surprisingly, iron supplementation led to a decrease in cell growth under acidic conditions (Figure S9). However, overloading the media with iron may have caused ferroptosis, rather than triggering a pro-survival action through stimulating iron-sulfur cluster biogenesis. Recently, ferroptosis regulators such as glutathione (GSH) peroxidase-4 (GPX4) and cystine/glutamate transporter SLC7A11 have been found to become upregulated under acidic conditions (Dierge et al., 2021). Consistent with this, treatment with the ferroptosis inhibitor ferrostatin-1 alleviated the growth defect caused by iron supplementation in SW1222 cells at low pHe (Figure S9).

### Xenografts of *NDUFS1* knockout cells show reduced tumor growth compared with wild-type cells

**Figure 7:**
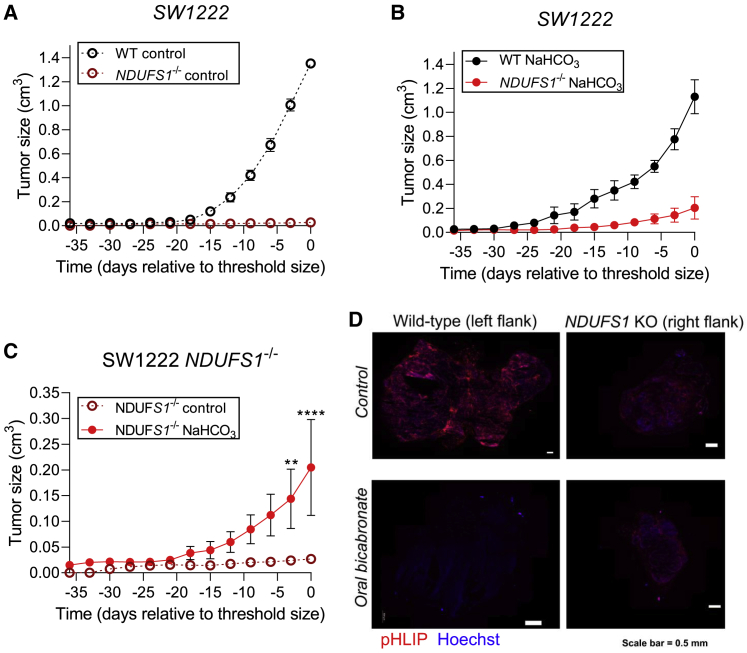
Xenografts of NDUFS1^−/−^ cells show reduced tumor growth compared with wild-type cells (A) SW1222 wild-type and *NDUFS1*^−/−^ tumor volume in mice receiving regular drinking water. Data represents paired measurements from injected with wild-type cells in their left flank and *NDUFS1*^−/−^ in their right flank (mean ± SEM of six animals). (B) SW1222 wild-type and *NDUFS1*^−/−^ tumor volume in mice receiving oral sodium bicarbonate treatment. Data represents paired measurements from injected with wild-type cells in their left flank and *NDUFS1*^−/−^ in their right flank (mean ± SEM of six animals). (C) Comparison of tumor volume between *NDUFS1*^−/−^ in control and sodium-bicarbonate-treated animals (mean ± SEM of six animals per group). Significance determined with two-way ANOVA using Šídák's multiple comparisons test (^∗^p < 0.05, ^∗∗^p < 0.01, ^∗∗∗^p < 0.001; ns, non-significant, p > 0.05). (D) Representative images of Cy5.5-conjugated pH-(low)-insertion peptide (pHLIP, red) and Hoechst-33342 (blue) in the fresh frozen tumor sections of wild-type and *NDUFS1*^−/−^ SW1222 xenografts in animals receiving oral bicarbonate or water (control). Scale bar, 500 μm. Note that only tiles containing the tumor were scanned.

We next evaluated OXPHOS inhibition as a strategy to reduce tumor growth *in vivo*. Wild-type and *NDUFS1*^−/−^ SW1222 cells were injected subcutaneously to the left and right flanks, respectively, of immunodeficient nude mice to establish paired xenografts. In the cohort of 12 mice, half were given 400 mM sodium bicarbonate in their drinking water *ad lib*, and the other half were allocated to the control group (access to water). Oral sodium bicarbonate has previously been shown to raise tumor pHe in mice by systemic buffer loading (Robey et al., 2009). In the control (water) group, growth of xenografts established from *NDUFS1*^−/−^ cells was completely abrogated, compared with wild-type cells that attained a humane endpoint size within a few weeks (Figure 7A). This finding demonstrates the importance of OXPHOS as a survival mechanism for under-perfused tumors. Mice in the bicarbonate treatment group injected with wild-type cells had a normal trajectory of tumor growth (Figure 7B), which was modestly slower than in the non-bicarbonate control group. Importantly, oral bicarbonate had a substantial stimulatory effect on *NDUFS1*^−/−^ xenografts (Figure 7C). Thus, raising systemic buffering rescues growth of *NDUFS1*^−/−^ (i.e., pHe-sensitive) cells *in vivo*. This finding also confirms that the effect of OXPHOS genetic ablation on tumor growth is a pHe-dependent phenomenon. We confirmed that oral bicarbonate raises tumor pHe by imaging Cy5.5-conjugated pH-low insertion protein (pHLIP) injected to mice prior to killing. A dispersed and strong pHLIP signal was detected in wild-type xenografts of control (water) animals, indicating acidic regions. pHLIP signal was reduced in tumors from mice that received oral bicarbonate, consistent with the effect of supplemented buffering (Figure 7D). pHLIP signal was very weak in *NDUFS1*^−/−^ xenografts in the control (water) group, which is consistent with minimal growth *in vivo*. Oral bicarbonate stimulated the growth of *NDUFS1*^−/−^ xenografts, which is expected to increase overall metabolic acid loading, but the effect of this on tumor pHe would be offset by higher buffering. Consistent with this, the pattern of pHLIP staining was diffuse (Figure 7D).

## Discussion

Here, we show that genes involved in mitochondrial energy metabolism facilitate cancer cell survival under acid stress. Ablation of these genes selectively reduces the growth of cancer cells at low pHe, with only a small or minimal impact at physiological pHe. This therefore represents a highly selective therapeutic strategy for targeting acidic tumor regions, without affecting surrounding normal tissues. Using an unbiased genetic screening approach, we identified components of the OXPHOS pathway, such as *NDUFS1*, as suitable targets for such therapeutic intervention. Furthermore, we report that genes involved in iron-sulfur cluster biogenesis are also essential for cell survival under acidic conditions. Whereas the function of glycolytic genes becomes dispensable at acidic conditions, cells become increasingly reliant on the availability of O_2._ These findings are consistent with previous reports showing that OXPHOS gene mRNA levels are upregulated at low pHe (Rohani et al., 2019). Other studies have also reported an inhibition of glycolysis under tumor acidosis (Corbet et al., 2016; Chen et al., 2008). When one source of energy (glycolysis) is limited, the acidic tumor becomes increasingly vulnerable to the inhibition of any remaining pathways (i.e., mitochondrial metabolism).

Our findings raise the possibility of therapeutically targeting OXPHOS in combination with acid stress as a potential cancer treatment option. In support of this, we identify several small-molecule drugs that inhibit mitochondrial metabolism and can, when titrated appropriately, kill cancer cells selectively at acidic pHe. By eliminating cells from acidic niches, it may also be possible to target the tumor’s ability to evade immune surveillance, and therefore also improve the efficacy of immunotherapies (Buck et al., 2017; Chang et al., 2013). One example of a drug that may achieve selective killing is rotenone, an insecticide and a potent inhibitor of complex I. However, due to its lipophilicity and ability to cross the blood-brain barrier, it has been reported to cause symptoms of Parkinson's disease in humans (Tanner et al., 2011). Although some OXPHOS inhibitors are toxic in humans, others are used widely as drugs for various conditions. For example, the complex III inhibitor atovaquone is currently used in the treatment of malaria and pneumocystis pneumonia, with tolerable side effects. Atovaquone was recently trialed in non-small cell lung cancer patients, where it showed an inhibitory effect on tumor hypoxia (Skwarski et al., 2021). Another case is pioglitazone, an inhibitor of iron-sulfur cluster metabolism, which is currently used as medication for type II diabetes. These examples demonstrate that certain inhibitors of mitochondrial metabolism could be used safely in human patients. Non-tumoral actions could be mitigated by appropriate chemical designs or delivery systems. Recently, pHLIP has been used as a delivery platform to specifically target the acidic tumor microenvironment. pHLIP-mediated genetic silencing of *CEACAM6* demonstrated therapeutic efficacy against lung adenocarcinoma in mice (Son et al., 2019). Similar strategies could be adapted to genetically target OXPHOS in acidic tumors. Further experiments are needed to test the response of OXPHOS inhibitors on a variety of cancer cell lines with different genetic backgrounds. The outcome of these experiments could highlight opportunities to target specific mutations, and further limit side effects.

Targeting OXPHOS as a therapeutic strategy is only effective in tumors with insufficient vascularization, since highly perfused tumors are unlikely to retain an acidic microenvironment. Some rapidly growing solid tumors have been shown to stimulate angiogenesis to improve the delivery of nutrients, which would also have the effect of washing away extracellular acidity. Inhibiting angiogenesis in these tumors, e.g., with anti-VEGF therapy, could act as a strategy to exacerbate tumor acidosis (Jain, 2014) and render the tumor more vulnerable to OXPHOS inhibitors.

### Limitations of the study

The findings of our study relate to the effects of acidosis, and it must be recognized that this is one of several chemical features of the tumor microenvironment. To investigate the cellular responses to acidity, our experimental strategy was to control pHe while keeping other variables (including O_2_ tension and substrate supply) constant. This necessarily reductionist approach allowed us to identify genes required for survival at low pHe. In tumors, the presence of additional influences, such as hypoxia, may affect the efficacy of these pro-survival genes, and therefore future experiments implementing various combinations of the key chemical signatures of tumors are warranted to seek synergies and antagonisms between pro-survival pathways. While clonal cell models have been used successfully in studies of cancer metabolism, their limitations should be noted. In particular, culture systems cannot fully recapitulate the tumor microenvironment and its dynamic responses to cellular activities. Tumor acidosis cannot develop without impaired blood perfusion, but this would also affect other environmental variables, notably oxygen tension, substrate provision, and waste product removal. Further validation of our findings will require *in vivo* interventions that will ascertain the extent to which genes required for survival at low pH are also critical for tumor growth in a more general context.

## STAR★Methods

### Key resource table

**Table:** 

REAGENT or RESOURCE	SOURCE	IDENTIFIER
**Antibodies**		
Rabbit polyclonal anti-NDUFS1	Thermo Fisher	PA5-22309; RRID: AB_11151879
HPR-labelled mouse monoclonal anti-GAPDH	ProteinTech	HRP-60004; AB_2737588

**Bacterial and virus strains**		

Toronto KnockOut (TKO) CRISPR Library v3	Addgene	90294, 125517
lentiCRISPR v2	Addgene	52961
5-alpha Competent E. coli (High Efficiency)	New England Biolabs	C2987H

**Chemicals, peptides and recombinant proteins**		

DMEM	Life technologies,	41965-039
Sodium bicarbonate-free DMEM	Sigma-Aldrich	D7777
Sodium bicarbonate and phenol red-free DMEM	Sigma-Aldrich	D5030
Foetal Bovine Serum	Merck Life Science	F9665-500ML
Penicillin-Streptomycin	Sigma-Aldrich	P0781
Sodium bicarbonate	Sigma-Aldrich	S5761
Sodium chloride	Sigma-Aldrich	S5653
Glutamine	Sigma-Aldrich	G7513
KAPA HiFi Hotstart ReadyMix	Roche	7958935001
AMpure XP	Beckman Coulter	A63880
Polybrene	Merck Life Science	H9268-5G
Puromycin	Santa Cruz	sc-108071A
Sulphorhodamine B	Sigma-Aldrich	230162-5G
Trichloroacetic acid	Merck Life Science	91230-100G
Acetic acid	Sigma-Aldrich	A6283-500ML
Tris Base	Sigma-Aldrich	T1503-1KG
Radioimmunoprecipitation assay (RIPA) buffer	Cell Signalling	9806S
Acrylamide	Geneflow Ltd	A2-0074
8-Hydroxypyrene-1,3,6-trisulfonic acid trisodium salt (HPTS)	Sigma-Aldrich	H1529
Tris(bipyridine)ruthenium(II) chloride (RuBPY)	Sigma-Aldrich	224758
Sodium pyruvate	Gibco	11360-070
D-(+)-Glucose	Sigma-Aldrich	G7021-1KG
2',7'-dichlorodihydrofluorescein diacetate (H_2_DCFDA)	Tocris	5935
Hoechst 33342	Invitrogen	H3570
5-(and-6)-carboxy SNARF-1 acetoxymethyl ester, acetate	Invitrogen	C1272
Rotenone	Sigma-Aldrich	R8875-1G
Atovaquone	Cayman Chemical Company	23802
Piericidin A	ChemCruz	Sc-202287
Deferoxamine (mesylate)	Cayman Chemical Company	14595
Deferiprone	ChemCruz	Sc-211220
Deguelin	ChemCruz	Sc-200657
Pioglitazone	ChemCruz	sc-204848
Metformin (hydrochloride)	Cayman Chemical Company	13118
NL-1	Fisher Scientific	502030328
Fe(II) sulfate heptahydrate	BDH	10112
Matrigel	Corning	356234
pH-(low)-insertion peptide (pHLIP)	CSBio C	N/A

**Critical commercial assays**		

Blood and Cell Culture DNA Maxi Kit	QIAGEN	13362
QIAquick Gel Extraction Kit	QIAGEN	28706X4
MinElute PCR Purification Kit	QIAGEN	28004
NextSeq 500/550 High Output Kit v2.5	Illumina	20024906
Bicinchoninic acid (BCA) protein assay kit	Thermo Fisher Scientific	23225

**Deposited data**		

Whole-genome CRISPR/Cas9 screen data	This paper	GEO: GSE195484
Survival curve fitting code	This paper	Mendeley Data: https://doi.org/10.17632/3x3fv6n6cz.1

**Experimental models: Cell lines**		

Human: SW480	Walter Bodmer's laboratory, University of Oxford	CCL-228
Human: SW1222	Walter Bodmer's laboratory, University of Oxford	N/A
Human: COLO320DM	Walter Bodmer's laboratory, University of Oxford	CCL-220
Human: Intestinal myofibroblasts	Walter Bodmer's laboratory, University of Oxford	N/A
Human: CCD18	Walter Bodmer's laboratory, University of Oxford	N/A

**Experimental models: Organisms/Strains**		

Female athymic Nude Crl:NU(NCr)-Foxn1nu mice	Charles River	N/A

**Oligonucleotides**		

gRNA sequences listed in Table S7	Invitrogen	N/A

**Recombinant DNA**		

lentiCRISPR v2	Addgene	52961
lentiCRISPR constructs with gRNA inserts for all oligonucleotides listed above		In this paper

**Software and algorithms**		
Fiji	ImageJ	N/A
Gen5 v.10	Biotek	N/A
MATLAB R2020b	Mathworks	N/A
MAGeCKFlute	(Wang et al., 2019)	N/A
Panther Classification System	https://doi.org/10.5281/zenodo.5080993	N/A
DrugZ	(Colic et al., 2019)	N/A

### Resource availability

#### Lead contact

Further information and requests for resources and reagents should be directed to and will be fulfilled by the lead contact, Pawel Swietach (pawel.swietach@dpag.ox.ac.uk).

#### Materials availability

Plasmids generated in this study are available upon request from the lead contact.

#### Data and code availability

• CRISPR/Cas9 screen DNA sequencing data have been deposited at GEO and are publicly available as of the date of publication. Accession numbers are listed in the key resource table. Microscopy data reported in this paper will be shared by the lead contact upon request.
• All original code has been deposited at Mendeley and is publicly available as of the date of publication. DOIs are listed in the key resource table.
• Any additional information required to reanalyze the data reported in this paper is available from the lead contact upon request.

### Experimental model or subject details

#### Cell lines and culture conditions

Human colorectal cancer cell lines SW480 (male patient), SW1222 (unknown sex) and COLO320DM (female patient) as well as intestinal myofibroblasts (unknown sex) and CCD18-Co fibroblasts (female patient) were obtained from Professor Walter Bodmer's laboratory at the Weatherall Institute of Molecular Medicine (WIMM), University of Oxford. Cells were cultivated using DMEM (Life technologies, Cat. No. 41965-039) (supplemented with 10% FBS and 1% PS) at 37°C with 5% CO_2_. Alternatively, cells were treated with NaHCO_3_-free DMEM (Sigma-Aldrich, Cat. No. D7777, supplemented with 10% foetal bovine serum (FBS), 1% Penicillin-Streptomycin solution (PS, 10 000 U/mL) containing various concentrations of NaHCO_3_, NaCl and drugs.

#### Animals

Female athymic Nude Crl:NU(NCr)-Foxn1nu mice were 12 weeks old before injection subcutanous with either SW1222 WT or SW1222 *NDUFS1*^−/−^ cells. Animals were randomly assigned to either control or sodium bicarbonate treatment groups. All animal procedures were carried out in accordance with national and institutional guidelines, with the approval of ethics and welfare board instructions, and with the authority of Home Office Project Licence PPL P01A04016.

### Method details

#### Alteration and monitoring of medium pH

Media were prepared by mixing NaHCO_3_-free Dulbecco's modified Eagle's medium (DMEM) (Sigma-Aldrich, Cat. No. D7777), supplemented with 10% FBS (Sigma-Aldrich) and 1% penicillin-streptomycin (PS) (100 U/mL penicillin, 100 μL/mL streptomycin; Sigma-Aldrich). Medium pH was set by adjusting [HCO_3_^-^], achieved by mixing various ratios of stocks containing either 44 mM NaHCO_3_ or 44 mM NaCl. This strategy ensures that osmolarity is constant. Medium pH was measured b Phenol Red absorbance at 430 nm and 560 nm using Cytation 5 imaging plate reader equipped with a CO_2_ gas controller (Biotek). Measurement were taken from 200 μL medium in a clear, flat-bottom 96-well plate (Costar) without lids at 37°C.

#### Genome-wide CRISPR/Cas9 screen

The genome-wide CRISPR screen was performed in the colorectal cancer cell line SW480 (ATCC CCL-228). Throughout the screen, cells were maintained in culture in 5% CO_2_ and at 37°C. 180 million cells were infected with the TKO v.3 library virus at a target MOI of 0.3, allowing for a representation of 675 cells infected with a given sgRNA. The screen was carried out in duplicate, independent experimental set-ups. Cells were maintained in DMEM medium with 10% FBS and 1% PS and were diluted to a concentration of 0.2 million/mL and 8ug/mL polybrene. The cell suspension was then placed in 15-cm dish with 15 mL of cells per dish (3 million cells). TKO v3 viral supernatant was prepared using HEK293T cells plated onto 15 cm dishes, the following day cells were transfected with the TKO v3 library, pMD2.G and psPAX2. The media was changed 6 hours post transfection and collected 48 and 72 hours post transfection. Lentiviral supernatant was spun at 2000 g for 5 minutes, filtered using a 0.45 μM CA filter, aliquoted and stored at −80°C. 100ul of virus suspension was added to each dish. After 48 h, the medium containing virus was replaced by fresh medium containing 3 μg/mL puromycin. Puromycin selection was carried out for four days. On day seven after infection, puromycin was removed and cells were cultivated in DMEM medium for 48 hours before start of the pHe treatment. On day nine after infection, the cells were harvested into two duplicate pools using trypsin and counted. 3 million cells per dish were seeded and were incubated with 32 mL medium (NaHCO_3_-free DMEM D7777) containing either 22 mM, 5.5 mM or 2.75 mM sodium bicarbonate (equilibrated in 5% CO_2_ to pHe 7.4, 6.9 and 6.63) supplemented with various concentrations of NaCl to maintain constant osmolarity. pH treatment was carried out in duplicate and 20 dishes were seeded for each condition. The remaining cells were washed in PBS and stored at −80°C (sample T0). The medium was replaced after three days and the cells were passaged after five days. Cells from each condition were trypsinised and re-seeded at 3 million cells/dish. Remaining cells were collected (in duplicate) and stored at −80°C (sample T5). Medium was replaced after an additional two days of incubation and the cells were harvested 11 days after start of the pH treatment. Cells from each condition (in duplicate) were counted, washed in PBS and stored at −80°C (sample T11). T11 samples were used for sequencing. Genomic DNA was extracted using the QIAGEN Blood and Cell Culture DNA Maxi Kit.

#### Library preparation and sequencing

Genomic DNA (gDNA) was purified from two infection replicates from each of the three pHe conditions (pHe 7.4, pHe 6.9 and pHe 6.63). PCR of gDNA to attach Illumina sequencing adapters and sample barcodes was performed using the TKO v3 protocol except that 2.5 μg of gDNA was used per 50 μL reaction. KAPA HiFi Readymix was used to amplify sgRNA-containing regions. PCR products amplified from the same gDNA sample were pooled, separated on a 2% agarose gel, and purified with the QIAquick Gel Extraction kit (Qiagen). Samples were further purified using Minelute PCR purification kit and AMpure XP. Samples were sequenced on a NextSeq 500 using a 75 cycles NextSeq 500/550 High Output Kit v2.5 (Illumina 20024906).

#### CRISPR/Cas9 screen data analysis

Data obtained by the genome-wide CRSPR screen was analysed using the MAGeCKFlute pipeline (liulab-mageck-0.5.9.2) to perform read-count mapping, normalization and QC, as well as to identify positively and negatively selected genes in the screens (Wang et al., 2019). Pairwise analyses (pHe 6.9 v 7.4 and pHe 6.6 v 7.4) were performed using the TKO v3 library as the reference. Each condition had duplicate datasets. As per the standard analysis pipeline, functions were executed to remove batch effects, normalize and correct for copy-number. Briefly, FASTQ files were downloaded from CRISPRCloud2 (CC2; http://crispr.nrihub.org/), merged, and processed by MAGeCK-VISPR. Mappability was 88%–90% for three conditions and their duplicates. Pairwise gene hits were identified by MaGeCK RRA. Quality-control and assignment of beta score levels were performed by FlutemMLE. Pathway enrichment was performed by KEGG. Datasets were analysed by DrugZ (github.com/hart-lab/drugz) to identify chemogenetic interactions, which identifies genetic perturbations that enhance or suppress drug activity (Colic et al., 2019).

Subsequent Gene Ontology (GO) analysis was performed using GO enrichment analysis (http://geneontology.org/). Relevant gene lists were compared to the reference gene set (*Homo sapiens* all genes). Fisher's exact test was applied to rank enriched biological processes. False discovery rate (FDR) refers to gene overrepresentation results, calculated by the Benjamini-Hochberg procedure.

#### Experimental follow up

Knockouts were made from SW480 cells (ATCC CCL-228), SW1222 and COLO320DM cells (ATCC CCL-220) using media conditions analogous to the CRISPR screen conditions: DMEM, 10% FBS, 1% Pen/Strep. sgRNA sequences were cloned into LentiCRISPR v.2 backbone as previously described (http://genome-engineering.org/gecko/wp-content/uploads/2013/12/lentiCRISPRv2-and-lentiGuide-oligo-cloning-protocol.pdf). Two sgRNA sequences were cloned for each gene, using sequences listed in the pooled TKO v.3 library (key resource table). Virus aliquots were prepared by the Virus Production Facility at WIMM, University of Oxford. Cells were plated in clear, flat-bottom 6-well plate at a density of 200,000 cells/well and transduced using a 500 μL aliquot of lentivirus carrying the LentiCRISPR v2 construct encoding for a sgRNA sequence targeting one individual gene. Polybrene was added at a concentration of 4 μg/mL. The 6-well plate was incubated for two days before puromycin (3 μg/mL) was added for selection, and cells were incubated for three days before the transduced cells were used for setting up further experiments. Infected cells were seeded at 4000 cells/well in 200 μL of media (in wells of 96-well plates). 24h after seeding, medium was replaced with medium of six different bicarbonate concentrations (2.75, 5.5, 11, 22, 33, and 44 mM). Cells were incubated for six days and cell survival was determined using the sulphorhodamine B assay. Non-infected cells were used as a control. Lentivirus pools of knockout cells are mixed populations of cells with different genomic edits and unedited cells. Therefore, all growth curves were performed within two weeks of infection. With longer time points, it is possible that hypomorph or unedited cells will out-compete loss-of-function mutations.

#### Cell growth analysis using sulforhodamine B (SRB) assay

Cells were seeded at densities of 2,500-8,000 cells per well on clear, flat-bottom 96-well plate (Costar) with a growth area of 0.32 cm^2^ per well. The following day, medium was replaced by culture media at six different pHe levels (as described above). Cells were incubated at 37°C with 5% CO_2_ for six days. Afterwards, the SRB assay was performed where cells were first fixed using 100 μL/well 10% trichloroacetic acid at 4°C for 60 minutes; the fixed cells were washed with H_2_O for four times and stained using 100 μL/well SRB (0.057% SRB in 1% acetic acid) for 30 minutes; residual SRB was removed by washing with 200 μL/well 1% acetic acid four times before 200 μL/well 10 mM Tris base was added to dissolve SRB. SRB absorbance was recorded at 520 nm using Cytation 5 imaging plate reader. All experiments were carried out in triplicate. Three independent repeats were performed for testing sgRNA knock-outs in SW1222 and COLO320DM cells, and four independent repeats were performed in SW480 cells.

#### pH_i_ measurements

Cells were plated in triplicate at 50,000-100,000 cells per well in black wall, flat coverslip bottom μ-plate 96-well plates with a growth area of 0.56 cm^2^ per well (Ibidi) and were left to attach overnight. They were then incubated in Phenol red-free media supplemented with cSNARF1-AM and the nuclear stain Hoechst-33342 (10 μg mL^−1^, Molecular Probes), for 15 min, and then replaced with medium of varying sodium bicarbonate concentration (twice). Images of fluorescence excited at 377 nm and collected at 447 nm (Hoechst-33342), and of fluorescence excited at 531 nm and collected at 590 nm and 640 nm (cSNARF1), were acquired using Cytation 5 imaging plate reader (Biotek) and its bespoke software. Images were acquired using a 10x objective. Measurements were performed in an atmosphere of 37°C and 5% CO_2_, established in the plate reader. Further analysis of the population distribution of pH data was performed using a MATLAB script. cSNARF1 fluorescence ratios were converted into pH_i_ using a calibration curve obtained through the nigericin method.

#### Immunoblotting

Samples were prepared by trypsinising and lysing the cells using radioimmunoprecipitation assay (RIPA) buffer. Protein concentration in the samples was measured using bicinchoninic acid (BCA) protein assay kit and adjusted using water. Samples were loaded onto a 10% acrylamide gel. The gel was run at 90 V for 15 minutes and at 120 V for 90 minutes. Afterwards, membrane transfer was performed at 90 V for 90 minutes. Primary antibody against NDUFS1 protein (ThermoFisher Cat. No. PA5-22309) and goat anti-rabbit secondary antibody were applied, and the membrane was visualised using horseradish peroxidase. Antibody binding of GAPDH protein was used as a loading control.

#### Medium pH and oxygen usage monitoring using RhuBP assay

Cells were cultured at high density (70,000 cells/well) in flat-bottom, black 96-well plates. To report extracellular pH and O_2_, media contained 2 μM HPTS (8-Hydroxypyrene-1,3,6-trisulfonic acid trisodium salt) and 50 μM RuBPY (tris(bipyridine)ruthenium(II) chloride), as described previously (Blaszczak et al., 2021). Media were based on DMEM (D5030) and contained 25 mM glucose, 10% FBS, 1% PS, 1 mM pyruvate, 1% glutamax and varying concentrations of HEPES and MES, as indicated, to vary buffering conditions. NaCl was added to a concentration that maintains overall osmolarity. Prior to measurements, each well was sealed with 150 μM mineral oil to restrict O_2_ ingress. HPTS and RuBPY fluorescence were monitored for 17 h using a Cytation 5 device (BioTek, Agilent, Winooski, VT, USA). Excitation was provided by a monochromator, and fluorescence emission was detected sequentially at five wavelengths, which were optimized for the dye combination used. Optimal settings on our system were excitation wavelengths of 400, 416, 450, 460, and 540 nm, and the corresponding emissions were 510, 510, 620, 510, and 580 nm. and dissolved in water at stocks of 4 and 100 mM, respectively. To maintain a consistent molar ratio of HPTS and RuBPY, stocks were mixed accordingly (1:1 v/v) and stored at −20°C.

#### Reactive oxygen species (ROS) detection

Cells were seeded at densities of 2,500-8,000 cells per well on black, flat-bottom 96-well plate (Costar) with a growth area of 0.32 cm^2^ per well. The following day, medium was replaced by culture media at six different pH levels (as described above). Cells were incubated at 37°C with 5% CO_2_ for six days. The medium was removed and cells were incubated with 10 μM 2′,7′-dichlorodihydrofluorescein diacetate (H_2_DCFDA) and 20 μM Hoechst 33342 in PBS for 30 min. Afterwards, cells were washed 3 times in PBS. Fluorescense was immediately recorded using Cytation 5 plater reader at ex 495 nm/em 520 nm for H_2_DCFDA and ex 361nm/em497 for Hoechst33342. ROS levels are expressed as H_2_DCFDA/Hoechst33342 fluorescence ratio, to account for variation in cell numbers.

#### Drug treatments

Rotenone, atovaquone, piericidin A, deferoxamine and deguelin were dissolved in DMSO and stored at −20°C. Pioglitazone, metformin, deferiprone (DFI), Fe(II) sulfate were dissolved in H_2_O and stored at −20°C.

#### Survival curve fitting

The relationship between cell survival and medium pH for different cell lines and gene knock-outs was analysed using an in-house script written in Matlab. Each relationship was fitted to a biphasic Hill-type curve with an activatory and inhibitory binding constant (K and Q), each characterized with a cooperativity, as well as a maximum growth G_max_. Overall, five independent variables were used. The peak of growth curve informed the optimal pHe. Data from individual repeats were pooled and pH_50_ values were obtained for individual gene knock-outs, representing the medium pH which leads to a 50% decrease in survival compared to the optimal medium pH (100%).

#### *In vivo* xenograft experiments

Female athymic Nude Crl:NU(NCr)-Foxn1nu mice were 12 weeks old before injection subcutanous with either SW1222 WT or SW1222 *NDUFS1*^−/−^ cells. Cells were resuspended in 100 μL of a 1:1 mixture of matrigel and serum-free DMEM medium before injection. Each mouse was injected with 2 million SW1222 WT cells on the left flank and 2 million SW1222 *NDUFS1*^−/−^ cells on the right flank. Six mice received oral treatment of 400 mM sodium bicarbonate, which was added to their drinkng water. A group of six control mice received regular drinking water. Mice were weighed and tumors were measured 3 times a week. At the end of the experiments, when tumors reached the size of the ethical endpoint, mice were injected with pH-(low)-insertion peptide (pHLIP) and Hoechst 33342 for 20 min. Afterwards, mice were sacrificed and tumors were processed for imaging. Images of tumor cryo-sections were acquired on a Leica DMi8 microscope using a HC PL APO 20x0.8 objective. Only tiles which contain biological structure were scanned. The remaining tiles were not scanned and have pixel values of zero by default.

### Quantification and statistical analysis

Data was analysed using GraphPad Prism 9. Data are represented expressed as mean ± S.E.M. Data was compared using unpaired t-test, one-way ANOVA or two-way ANOVA, as indicated in the figure legends. p values of <0.05 were considered significant. ^∗^ = p < 0.05, ^∗∗^ = p < 0.01, ^∗∗∗^ = p < 0.001. Information on biological replicates (N) and significance (p values) of individual tests can be found in figure legends.

## Supplemental information

Document S1. Figures S1–S9, Tables S1, and S2–S6

Table S2: 181 gRNAs significantly enriched in pH 6.6 versus pH 7.4 conditions, related to Figures 1 and 3

Document S2. Article plus supplemental information
