# CRISPR-Cas9 screen identifies oxidative phosphorylation as essential for cancer cell survival at low extracellular pH

**DOI:** 10.1016/j.celrep.2024.114499

**Published:** 2024-07-02

**Authors:** Johanna Michl, Yunyi Wang, Stefania Monterisi, Wiktoria Blaszczak, Ryan Beveridge, Esther M. Bridges, Jana Koth, Walter F. Bodmer, Pawel Swietach

## Main text

(Cell Reports *38*, 110493; March 8, 2022)

In the originally published version of the paper, one funder was accidently omitted. This work was additionally supported by Marie Skłodowska-Curie Innovative Training Network (#813834 “pHioniC” H2020-MSCA-ITN-2018). The authors apologize for this oversight.

