# Supplementary material for: CRISPR-Cas9 screen identifies oxidative phosphorylation as essential for cancer cell survival at low extracellular pH
Source: Cell Rep. 2022 Mar 8;38(10):110493. doi: 10.1016/j.celrep.2022.110493 (PMC8924371; doi:10.1016/j.celrep.2022.110493)

Cell Reports, Volume *38*

Supplemental information

CRISPR-Cas9 screen identifies oxidative phosphorylation as essential for cancer cell survival at low extracellular pH

Johanna Michl, Yunyi Wang, Stefania Monterisi, Wiktoria Blaszczak, Ryan Beveridge, Esther M. Bridges, Jana Koth, Walter F. Bodmer, and Pawel Swietach

Table S1: 51 gRNAs significantly enriched in pH 7.4 vs. pH 6.6 conditions. Related to Figures 1 and 2.

GENE	Z-score	Normalised Z-score	p-value	Rank	FDR
NDUFS1	-26.24	-6.95	1.79E-12	1	3.23E-08
PRDX6	-25.65	-6.79	5.43E-12	2	4.90E-08
COX8A	-25.2	-6.68	1.24E-11	3	7.44E-08
TMED7	-23.83	-6.31	1.38E-10	4	6.23E-07
PRDX1	-20.3	-6.21	2.72E-10	5	9.81E-07
NDUFS2	-22.34	-5.91	1.68E-09	6	5.05E-06
NDUFA11	-22.07	-5.84	2.59E-09	7	6.69E-06
NDUFC1	-21.28	-5.63	8.86E-09	8	2.00E-05
C6orf136	-21.01	-5.56	1.35E-08	9	2.70E-05
LIAS	-19.93	-5.27	6.74E-08	10	0.000122
NDUFAF6	-19.64	-5.19	1.02E-07	11	0.000168
TM9SF3	-19.34	-5.11	1.57E-07	12	0.000236
NDUFB10	-18.08	-4.78	8.76E-07	13	0.00122
IBA57	-17.9	-4.73	1.11E-06	14	0.00143
TIMMDC1	-17.82	-4.71	1.24E-06	15	0.00149
NDUFA6	-17.56	-4.64	1.74E-06	16	0.00196
NFU1	-17.5	-4.62	1.87E-06	17	0.00199
NDUFB8	-17.16	-4.53	2.89E-06	18	0.00289
PTEN	-17.12	-4.52	3.06E-06	19	0.0029
PDSS2	-17.07	-4.51	3.26E-06	20	0.00293
GLRX5	-17.03	-4.5	3.42E-06	21	0.00293
COX5B	-16.94	-4.47	3.83E-06	22	0.00293
NDUFB6	-16.93	-4.47	3.88E-06	23	0.00293
NDUFA9	-14.66	-4.47	3.90E-06	24	0.00293
NDUFA10	-16.74	-4.42	4.87E-06	25	0.00352
NDUFB2	-16.68	-4.4	5.29E-06	26	0.00353
NDUFB11	-16.67	-4.4	5.32E-06	27	0.00353
NDUFV2	-16.65	-4.4	5.47E-06	28	0.00353
COQ2	-16.39	-4.33	7.51E-06	29	0.00468
TMEM261	-15.76	-4.16	1.58E-05	30	0.00947
NDUFAF4	-15.74	-4.16	1.63E-05	31	0.00947
NDUFS5	-15.16	-4	3.15E-05	32	0.0178
SLC9A1	-15.11	-3.99	3.32E-05	33	0.0182
NDUFA2	-15.07	-3.98	3.47E-05	34	0.0184
LacZ	-73.54	-3.96	3.73E-05	35	0.0192
TMX2	-14.72	-3.88	5.12E-05	36	0.0253
COX6B1	-14.71	-3.88	5.19E-05	37	0.0253
CNOT4	-14.62	-3.86	5.71E-05	38	0.0271
NDUFS3	-14.56	-3.84	6.14E-05	39	0.0284
LIPT1	-14.46	-3.81	6.85E-05	40	0.0309
NDUFA1	-13.97	-3.68	0.000115	41	0.0506
NDUFA5	-13.91	-3.67	0.000122	42	0.0526
COX6A1	-12.02	-3.66	0.000126	43	0.0528

ATG14	-13.65	-3.6	0.000159	44	0.0633
NDUFAF3	-13.65	-3.6	0.00016	45	0.0633
ATP5B	-13.64	-3.6	0.000161	46	0.0633
DCP2	-13.57	-3.58	0.000173	47	0.0659
ISCA2	-13.56	-3.57	0.000175	48	0.0659
FDX1	-13.37	-3.52	0.000213	49	0.0785
DLD	-13.32	-3.51	0.000222	50	0.0802
HPGD	-13.08	-3.45	0.000283	51	0.1

Table S3: Gene Ontology terms for pathways that are selectively essential at pHe 6.6 (red) or pHe 7.4 (blue). Gene Ontology analysis was performed using GO enrichment analysis (http://geneontology.org/). False discovery rate (FDR) refers to gene overrepresentation results, calculated by the Benjamini-Hochberg procedure. Related to Figure 1.

**Gene ontology biological process essential at pHe 6.6**	**Genes**	**FDR**
aerobic electron transport chain	23	8.2E-37
mitochondrial respiratory chain complex I assembly	22	8.2E-37
ATP synthesis coupled electron transport	23	1.5E-36
NADH dehydrogenase complex assembly	22	1.6E-36
mitochondrial ATP synthesis coupled electron transport	23	1.9E-36
respiratory electron transport chain	23	5.7E-35
oxidative phosphorylation	23	2.0E-34
mitochondrial respiratory chain complex assembly	22	7.4E-34
electron transport chain	24	2.4E-33

**Gene ontology biological process essential at pHe 7.4**	**Genes**	**FDR**
ribonucleoprotein complex biogenesis	51	3.7E-35
ncRNA processing	48	5.2E-33
ribosome biogenesis	43	4.3E-32
ncRNA metabolic process	49	3.9E-31
cellular nitrogen compound metabolic process	103	1.1E-30
nucleobase-containing compound metabolic process	92	7.3E-30
RNA processing	58	5.8E-29
heterocycle metabolic process	93	1.4E-28

Table S4: 43 gRNAs significantly enriched in pH 7.4 vs. pH 6.9 conditions. Related to Figures 1 and 2.

GENE	Z-score	Normalised Z-score	p-value	Rank	FDR
NDUFS1	-23.95	-7.49	3.38E-14	1	6.10E-10
C6orf136	-19.96	-6.23	2.29E-10	2	2.06E-06
PET117	-18.84	-5.88	2.06E-09	3	1.24E-05
COX8A	-17.33	-5.4	3.30E-08	4	0.000149
NDUFA6	-17.12	-5.33	4.80E-08	5	0.000173
NDUFS2	-16.91	-5.27	6.87E-08	6	0.000207
NDUFAF6	-16.72	-5.21	9.52E-08	7	0.000241
SLC31A1	-16.65	-5.19	1.07E-07	8	0.000241
NDUFA11	-16.42	-5.11	1.57E-07	9	0.000315
TMEM261	-16.11	-5.01	2.67E-07	10	0.000482
SLC39A10	-15.43	-4.8	7.92E-07	11	0.0013
TIMMDC1	-15.15	-4.71	1.24E-06	12	0.00186
NDUFB10	-14.76	-4.59	2.23E-06	13	0.00277
NDUFS3	-14.74	-4.58	2.30E-06	14	0.00277
NDUFB2	-14.74	-4.58	2.30E-06	15	0.00277
NDUFB8	-14.21	-4.42	5.04E-06	16	0.00562
COQ2	-14.18	-4.4	5.29E-06	17	0.00562
FTSJ2	-14.12	-4.39	5.76E-06	18	0.00578
LRPPRC	-14.08	-4.37	6.09E-06	19	0.00579
ATP5B	-13.86	-4.3	8.40E-06	20	0.00758
IBA57	-13.75	-4.27	9.83E-06	21	0.00845
MRPL53	-13.64	-4.24	1.14E-05	22	0.00935
COX5B	-13.61	-4.22	1.20E-05	23	0.00938
RTN4IP1	-13.48	-4.18	1.43E-05	24	0.0107
NDUFA9	-11.57	-4.15	1.70E-05	25	0.0121
NDUFB6	-13.33	-4.14	1.77E-05	26	0.0121
NME6	-13.31	-4.13	1.81E-05	27	0.0121
CDH7	-13.18	-4.09	2.17E-05	28	0.014
NDUFV2	-12.92	-4.01	3.09E-05	29	0.0193
NDUFA2	-12.78	-3.96	3.69E-05	30	0.0222
ACAD9	-12.7	-3.94	4.14E-05	31	0.0241
NDUFAF4	-12.65	-3.92	4.38E-05	32	0.0247
MRPS16	-12.53	-3.88	5.16E-05	33	0.0278
PDSS2	-12.52	-3.88	5.24E-05	34	0.0278
COA5	-12.42	-3.85	5.97E-05	35	0.0308
NDUFS5	-12.24	-3.79	7.45E-05	36	0.0374
SUPV3L1	-12.07	-3.74	9.24E-05	37	0.0451
NAP1L4	-12.02	-3.72	9.83E-05	38	0.0467
COA6	-11.73	-3.63	0.000141	39	0.0639
MRPS18B	-11.73	-3.63	0.000141	40	0.0639
NDUFA1	-11.67	-3.61	0.000153	41	0.0672
NDUFC1	-11.59	-3.59	0.000167	42	0.0717
SLC4A7	-11.34	-3.51	0.000226	43	0.0948

Table S5: 43 gRNAs significantly enriched in pH 6.9 vs. pH 7.4 conditions. Related to Figures 1 and 3.

GENE	Z-score	Normalised Z-score	p-value	Rank	FDR
NOP9	15.9	5.11	1.64E-07	1	0.00148
TADA1	15.59	5.01	2.77E-07	2	0.00148
ALDOA	15.48	4.97	3.29E-07	3	0.00148
DOLK	15.43	4.96	3.56E-07	4	0.00148
DHX35	15.35	4.93	4.1E-07	5	0.00148
WDR92	14.76	4.74	1.04E-06	6	0.00314
PAICS	14.49	4.66	1.58E-06	7	0.00408
SLC16A3	13.85	4.46	4.14E-06	8	0.00846
PGK1	13.84	4.45	4.22E-06	9	0.00846
NDNL2	13.39	4.31	8.1E-06	10	0.0134
MED12	13.38	4.31	8.18E-06	11	0.0134
XRCC6	13.3	4.28	9.18E-06	12	0.0138
BTAF1	13.2	4.25	1.06E-05	13	0.0144
THG1L	13.16	4.24	1.11E-05	14	0.0144
TXNL4B	13.09	4.22	1.24E-05	15	0.0148
EXOSC1	13.05	4.2	1.31E-05	16	0.0148
TEN1	12.68	4.09	2.18E-05	17	0.0231
WRB	12.62	4.07	2.38E-05	18	0.0239
TSEN54	12.54	4.04	2.65E-05	19	0.0252
PTPMT1	12.36	3.99	3.36E-05	20	0.0303
FNTB	12.3	3.97	3.64E-05	21	0.0307
DDX59	10.63	3.96	3.79E-05	22	0.0307
DSCC1	12.25	3.95	3.91E-05	23	0.0307
DPH5	12.07	3.89	4.93E-05	24	0.0371
CDC123	11.94	3.85	5.85E-05	25	0.0392
EIF3A	11.9	3.84	6.12E-05	26	0.0392
INTS10	11.89	3.84	0.000062	27	0.0392
ARMC7	11.88	3.84	6.27E-05	28	0.0392
NSMCE1	11.88	3.83	0.000063	29	0.0392
RTCB	11.73	3.79	7.64E-05	30	0.046
PPP4C	11.5	3.71	0.000102	31	0.0576
TSC1	11.5	3.71	0.000102	32	0.0576
WDR7	11.25	3.63	0.000139	33	0.0747
ASNA1	11.22	3.63	0.000144	34	0.0747
MGEA5	11.22	3.62	0.000145	35	0.0747
METTL3	11.16	3.61	0.000154	36	0.0774
HSPA14	11.1	3.59	0.000167	37	0.0814
WDR61	11.04	3.57	0.000179	38	0.0831
DENR	11.04	3.57	0.000179	39	0.0831
SLC35B1	10.95	3.54	0.0002	40	0.0903
YKT6	10.89	3.52	0.000214	41	0.0922
TSC2	10.89	3.52	0.000216	42	0.0922
TEX10	10.87	3.52	0.00022	43	0.0922

Figure S1: Relationship between extracellular and intracellular pH in SW1222, SW480 and COLO320DM cells, loaded with cSNARF1 and Hoechst 33342 and imaged using a plate imaging microscope (mean±SEM of 3 independent repeats, with three technical replicates each). Related to Figure 1.

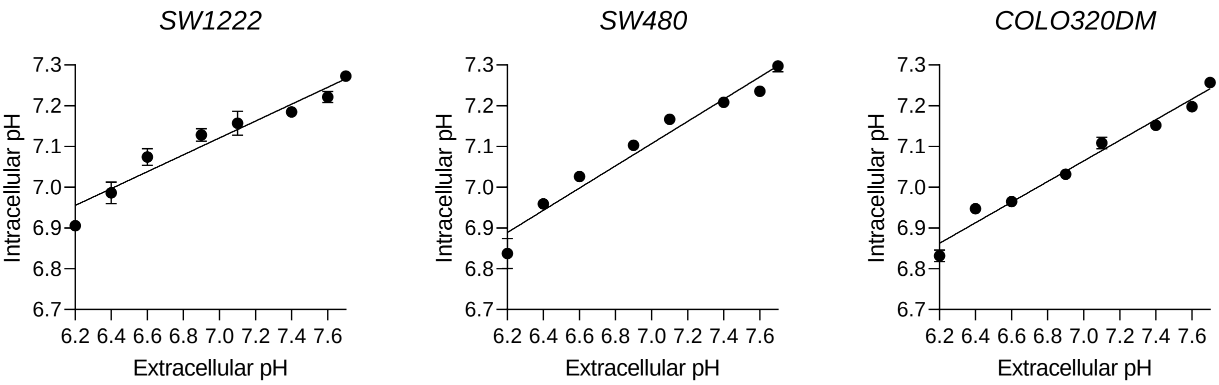

Table S6: gRNA sequences used for CRISRPR/Cas9 screen hit validation. Related to Figures 2 and 3.

gRNA name	Supplier	Sequence
C6orf136 gRNA1	Invitrogen	ATCATGTACCAGCCCAGCCG
C6orf136 gRNA2	Invitrogen	GAACCACCCATGCTGAGCTG
LIAS gRNA1	Invitrogen	GAGGAAATGTCTCTACGCTG
LIAS gRNA2	Invitrogen	TAAAGACAGAGATTCCCATG
TMED7 gRNA1	Invitrogen	AGAACCACCACAACTCGTGT
TMED7 gRNA2	Invitrogen	TCTGGTGGTTAGCATAGGGC
PRDX6 gRNA1	Invitrogen	ATCACCGTCGCCATGCCCGG
PRDX6 gRNA2	Invitrogen	TTTGAGGCCAATACCACCGT
NDUFS1 gRNA1	Invitrogen	TAGAATGTATGCCTACTTGG
NDUFS1 gRNA2	Invitrogen	TCACAAATAGGACAGTCCAA
PTEN gRNA1	Invitrogen	GGTTTGATAAGTTCTAGCTG
PTEN gRNA2	Invitrogen	TCATCTGGATTATAGACCAG
NFU1 gRNA1	Invitrogen	ACAGTAATATACCTAGCCAG
NFU1 gRNA2	Invitrogen	GGTGTTTCCTCAGTAACCAG
IBA57 gRNA1	Invitrogen	TAGCAGGAAGGGCGCCGCGT
IBA57 gRNA2	Invitrogen	GCGGCCGCAGGACTCGGAAG
TM9SF3 gRNA1	Invitrogen	ACGCCAAGAGCGCCAGGCAG
TM9SF3 gRNA2	Invitrogen	TCTGGGAGAAGCACTTCAAG
DOLK gRNA1	Invitrogen	GTGGCGGCTACATAGAGCAG
DOLK gRNA2	Invitrogen	GCCAGGGTAGGACCACACCA
PTPMT1 gRNA1	Invitrogen	TGGCGCGTCAAGCTCCGCAA
PTPMT1 gRNA2	Invitrogen	TACAGGACGAGAACGTGCGC
NOP9 gRNA1	Invitrogen	TCAATGCTGACAGCGCCCGG
NOP9 gRNA2	Invitrogen	TACCAGATGATTGGGAACCA
TADA1 gRNA1	Invitrogen	GGCCAAGAAGAACTTAAGCG
TADA1 gRNA2	Invitrogen	ACTGGGCTAACCTAAAGCTG
FLCN gRNA1	Invitrogen	AATGAGGACAGTCCTGGCCA
FLCN gRNA2	Invitrogen	ATCAAGGACAGCCTGGCCAG
PAICS gRNA1	Invitrogen	TAGGATAATGGCGACAGCTG
PAICS gRNA2	Invitrogen	AGGATAATGGCGACAGCTGA
RTCB gRNA1	Invitrogen	GTCCACAGTGATCTTACCTG
RTCB gRNA2	Invitrogen	GTCGGGTTTGACATCAACTG
NDNL2 gRNA1	Invitrogen	AAAGCCTCACAGTACTGCGC
TEN1 gRNA1	Invitrogen	GGCCTGCACTAACCTCCCAG
TEN1 gRNA2	Invitrogen	TGGCCTGCACTAACCTCCCA
FAM96B gRNA1	Invitrogen	GGTTCCGCGATGGTAGGCGG
FAM96B gRNA2	Invitrogen	GATGGACAGACCAATAAGGG
COX8A gRNA1	Invitrogen	GCTTGACAGGCTCGGCCCGG

COX8A gRNA2	Invitrogen	GATCCATTCGTTGCCGCCGG
CHTF8 gRNA1	Invitrogen	AGACCTACATTACACCACTG
CHTF8 gRNA2	Invitrogen	TGTAATGTAGGTCTCCCAGG
GAPDH gRNA1	Invitrogen	TTCCACTCACTCCTGGAAGA
ALDOA gRNA1	Invitrogen	CATTGGCACCGAGAACACCG
ALDOA gRNA2	Invitrogen	AATGGCGAGACTACCACCCA
WDR91 gRNA1	Invitrogen	GGTGGACTAGGAATTGGAGA

Figure S2: Experimental validation of negative selection screen hits in SW1222 KO cells. Growth (expressed as SRB absorbance) was assayed at 6-day as a function of pHe (mean±SEM of 3 independent repeats, with three technical replicates each). Related to Figure 2D.

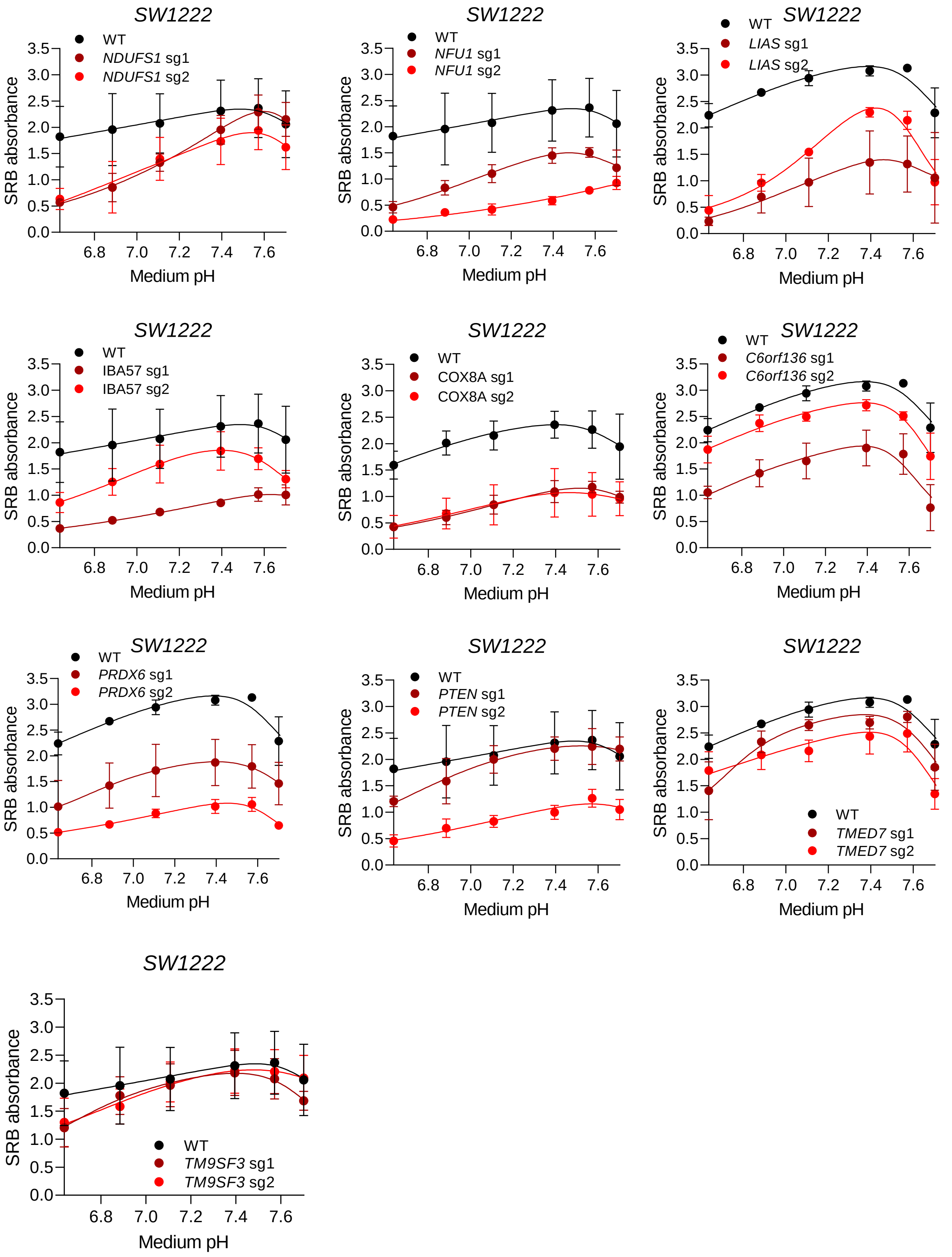

Figure S3: Experimental validation of negative selection screen hits in SW480 KO cells. Growth (expressed as SRB absorbance) was assayed at 6-day as a function of pHe (mean±SEM of 3 independent repeats, with three technical replicates each). Related to Figure 2C.

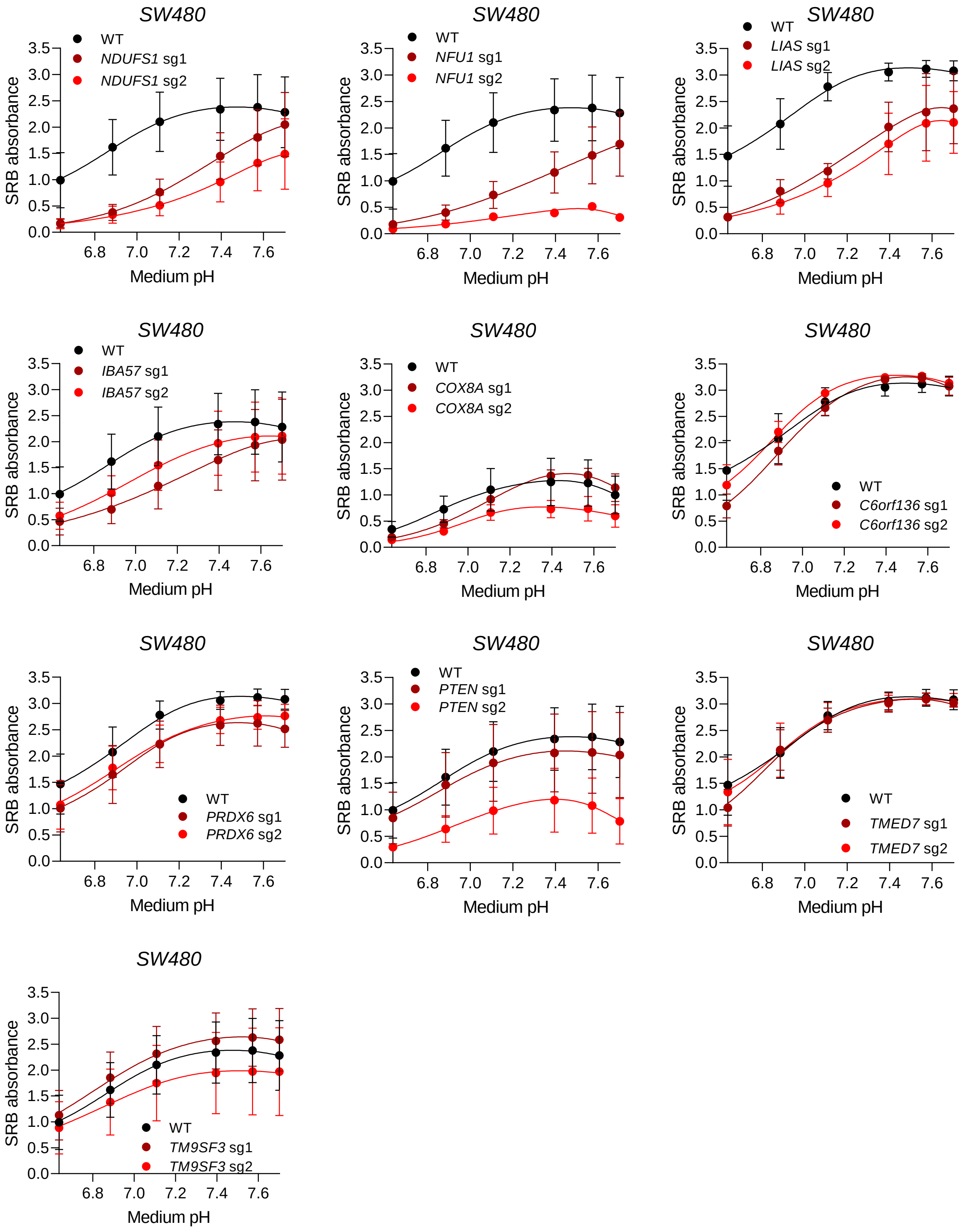

Figure S4: Experimental validation of positive selection screen hits in SW480 KO cells. Growth (expressed as SRB absorbance) was assayed at 6-day as a function of pHe (mean±SEM of 3 independent repeats, with three technical replicates each). Related to Figure 3C.

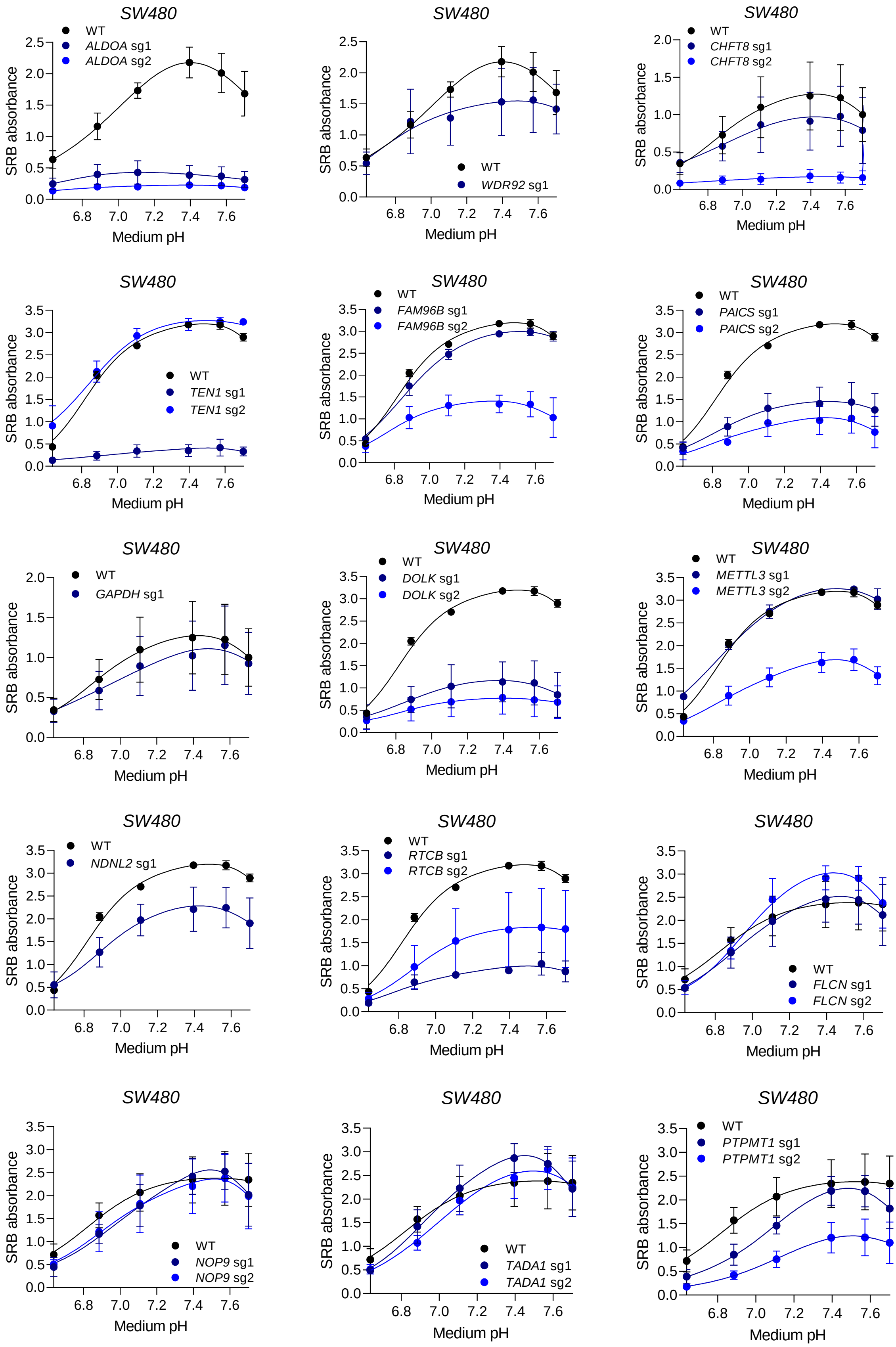

Figure S5: Experimental validation of positive selection screen hits in COLO320DM KO cells. Growth (expressed as SRB absorbance) was assayed at 6-day as a function of pHe (mean±SEM of 3 independent repeats, with three technical replicates each). Related to Figure 3D.

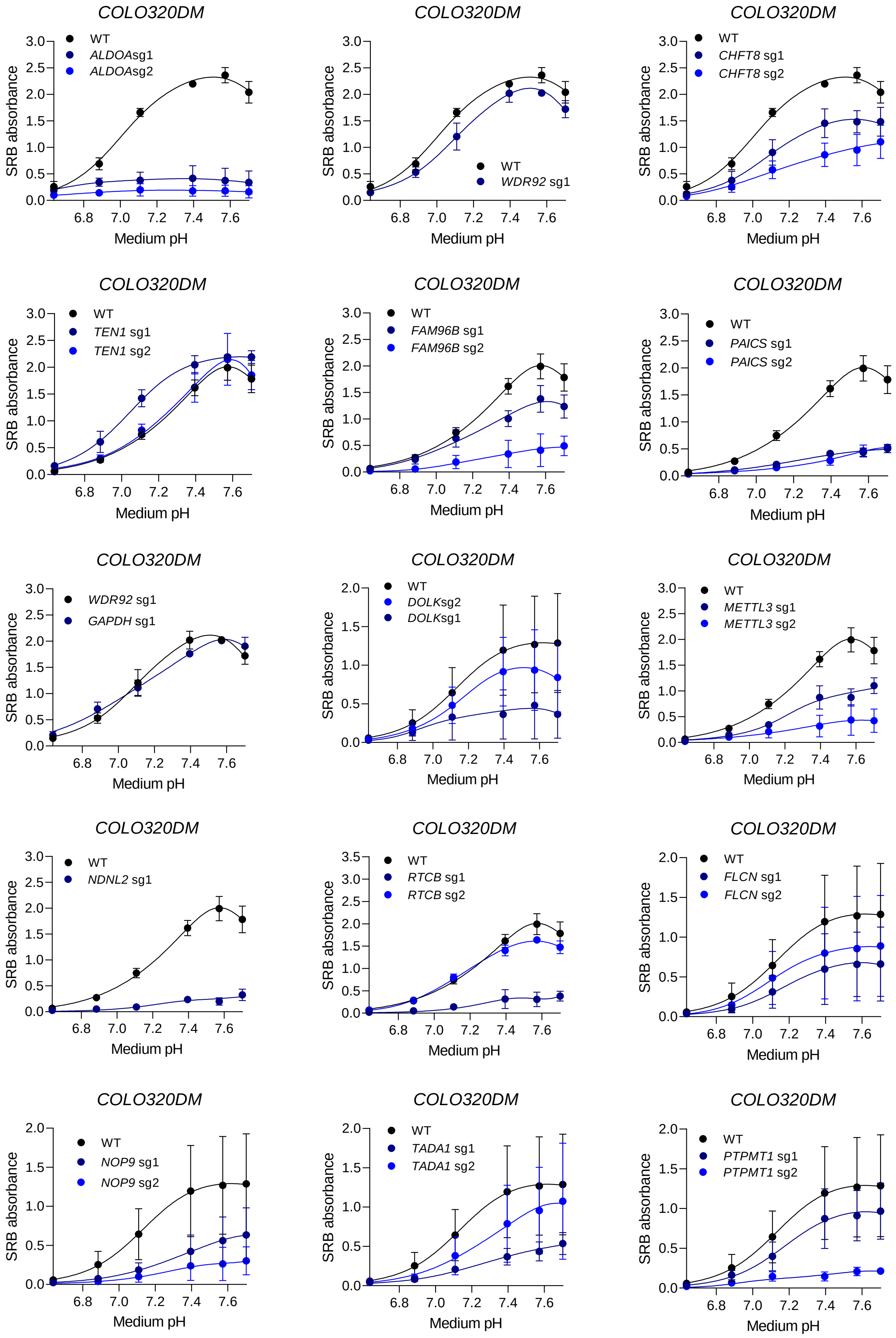

Figure S6: Reactive oxygen species (ROS) levels in SW1222 wild-type and SW1222 *NDUFS1*$^{-/-}$ cells cultured for six days at varying pHe. ROS levels expressed as H_2DCFDA fluorescence normalised to Hoechst 33342 fluorescence (mean±SEM of 5 independent repeats, with three technical replicates each). Related to Figure 5.

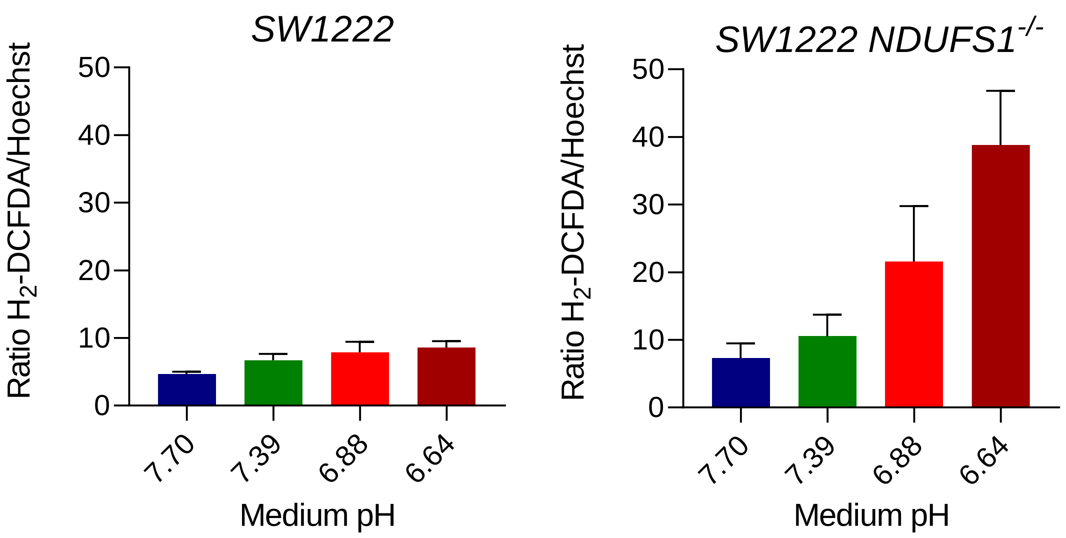

Figure S7: Normalised growth rates (measured by SRB absorbance) of SW480 and SW1222 and cells cultured for six days with 10 nM piericidin A, 50 nM deguelin, 500 µM metformin or vehicle. Data are plotted as relative cell growth normalized to optimum pHe (mean n=3 independent repeats ± SEM (carried out in technical triplicates)). Related to Figure 5. Fluorimetric assay for H^+ production and O_2 consumption under culture conditions (see Blaszczak et al, 2021). Cells were seeded at 100,000 per well and incubated in CO_2-free atmosphere for 17 h in a plate reader that registers pH (HPTS) and O_2 (RuBPY). The pH and O_2 time courses are converted to H^+ production and O_2 consumption (plots with shaded backgrounds). Measurements expressed relative to cell-free blanks (yellow). Some wells included OXPHOS inhibitors. The total concentration of drugs added was 10 µM rotenone, 10 µM deguelin, 0.1 µM piericidin A, 30 µM ATQ. A layer of mineral oil (150 µL) was used to cover the culture medium (100 µL), and is required to produce a closed environment for O_2 consumption measurements. Mean of n=3 independent repeats ± SEM (carried out in technical triplicates).

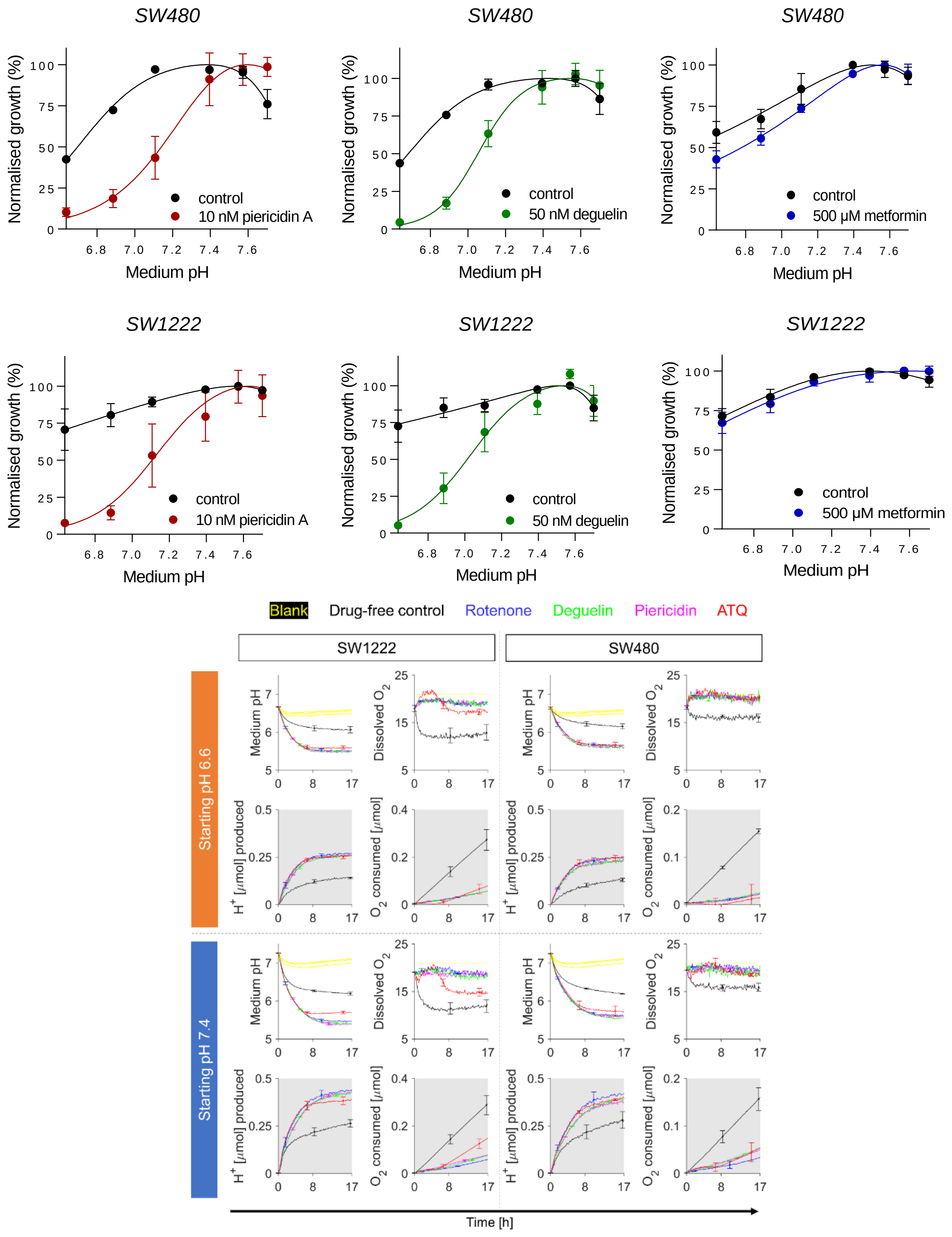

Figure S8: Effect of OXPHOS inhibitors on the pH-dependence of growth in cells that had been adapted to acidic conditions. SW480 and SW1222 cells were first adapted to pH 6.3 (reduced [HCO_3^-] and 10 mM MES) for one week, and in parallel, time-matched controls were kept at pH 7.4. Afterwards, cells were cultured for six days with 10 nM rotenone,10 µM ATQ or vehicle at the pHe values indicated on the x-axis. Growth was measured in terms of normalized SRB absorbance (mean n=3 independent repeats ± SEM (carried out in technical triplicates)). Normalised growth rates (measured by SRB absorbance) of intestinal myofibroblasts and CCD18 colonic fibroblasts cultured for six days with 10 nM rotenone, 10 µM ATQ or vehicle. Data are plotted as relative cell growth normalized to optimum pHe (mean n=3 independent repeats ± SEM (carried out in technical triplicates)). Related to Figure 5.

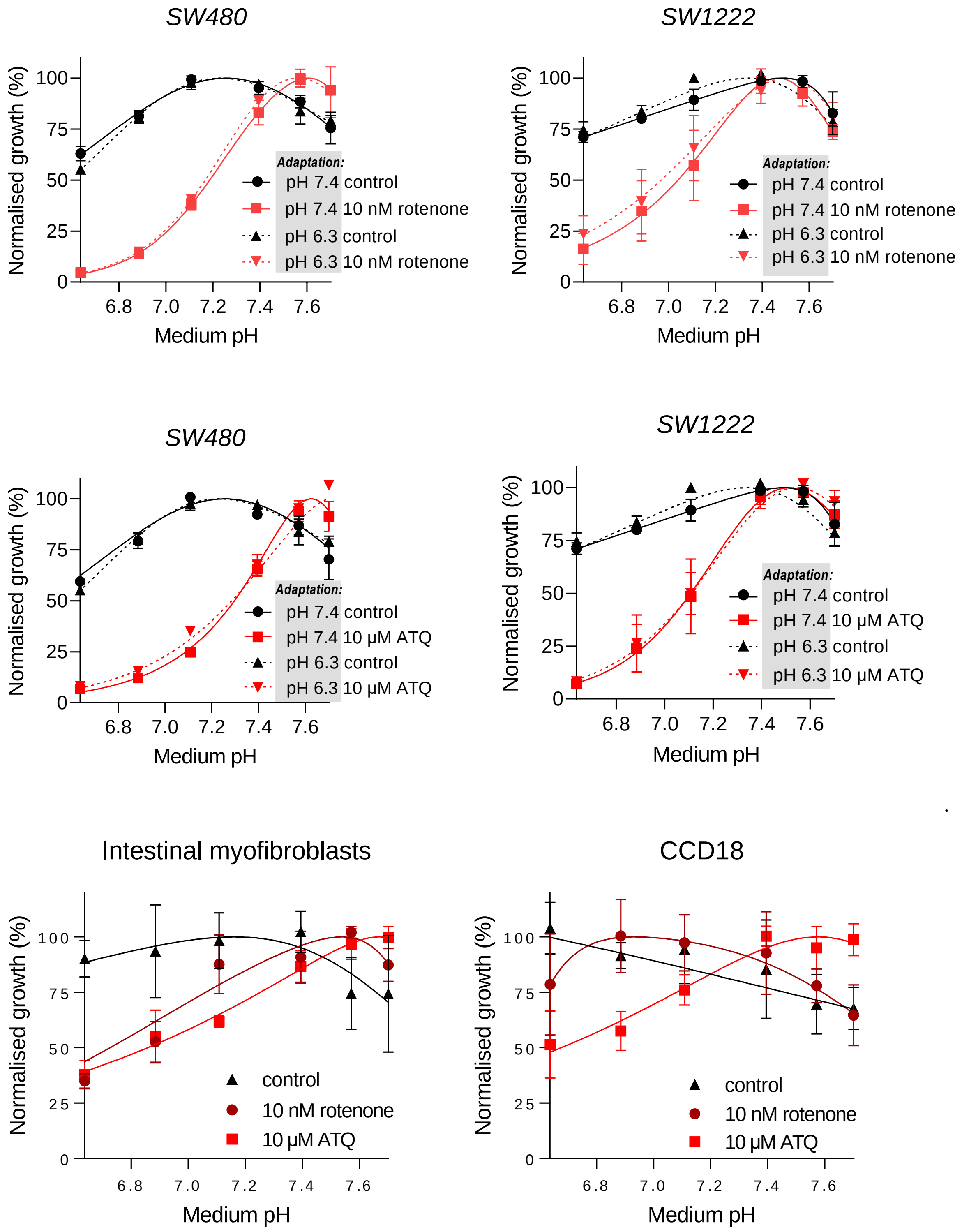

Figure S9: Normalised growth rates (measured by SRB absorbance) of SW480 and SW1222 and cells cultured for six days with 1 µM deferoxamine (DFO) 3 µM deferiprone (DFI), with 500 µM Fe(II) sulfate, 500 µM Fe(II) + 1 µM ferrostatin-1 or vehicle. Data are plotted as relative cell growth normalized to optimum pHe (mean n=3-5 independent repeats ± SEM (carried out in technical triplicates)). Related to Figure 6.

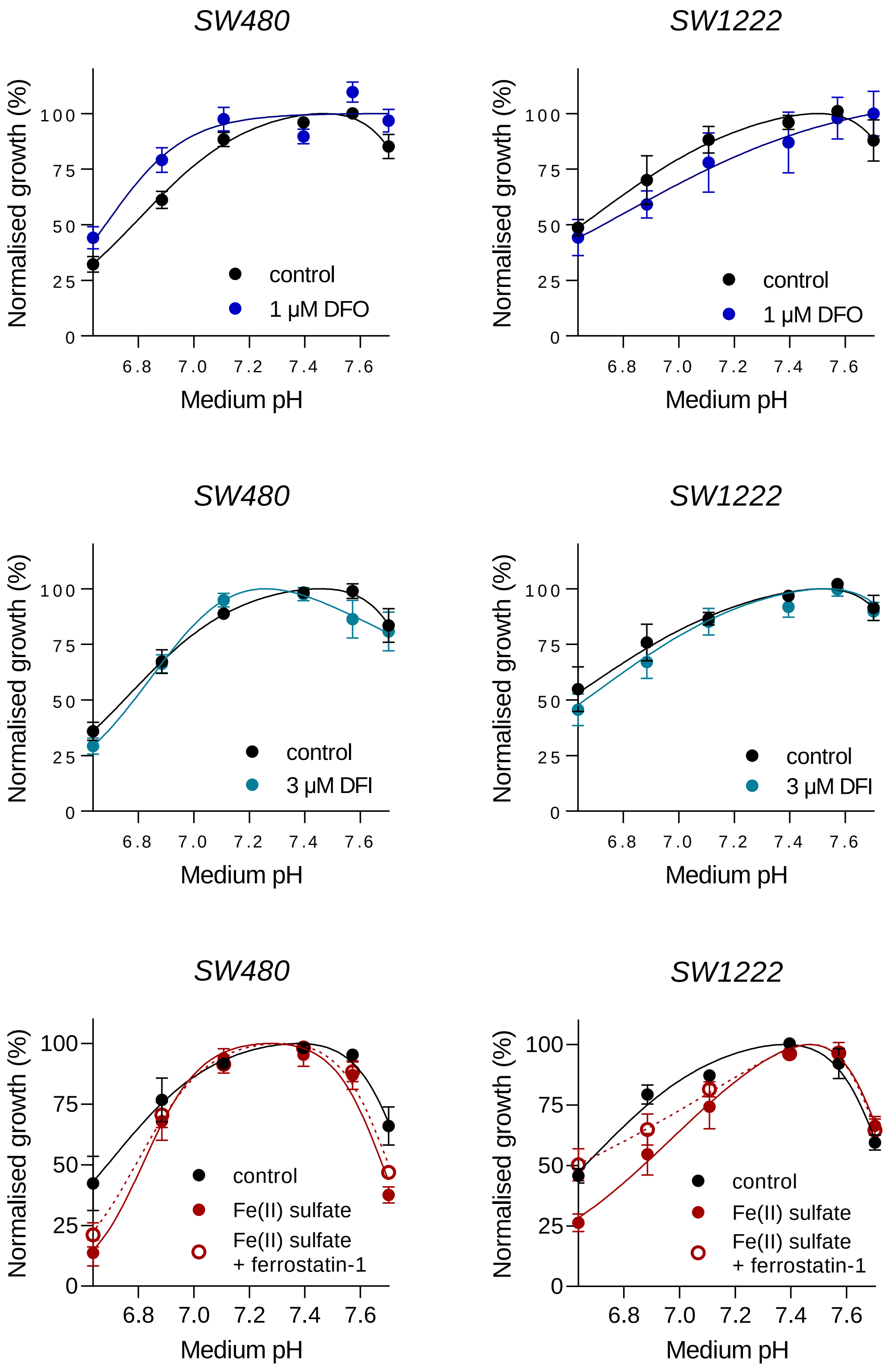

Supplement: Document S1. Figures S1–S9, Tables S1, and S2–S6 [file mmc1.pdf]
